# Health, economic and social impacts of the Brazilian cash transfer program on the lives of its beneficiaries: a scoping review

**DOI:** 10.1186/s12889-024-20046-2

**Published:** 2024-10-14

**Authors:** Júlia Magalhães, Carolina Ziebold, Sara Evans-Lacko, Alicia Matijasevich, Cristiane Silvestre Paula

**Affiliations:** 1 Human Developmental Sciences Graduate Program and Mackenzie Center for Research in Childhood and Adolescence, Mackenzie Presbyterian University, São Paulo, SP Brazil; 2 Department of Psychiatry, Federal University of Sao Paulo, São Paulo, SP Brazil; 3 Care Policy and Evaluation Centre, London School of Economics and Political Science, London, Great Britain; 4 Departamento de Medicina Preventiva, Faculdade de Medicina FMUSP, Universidade de São Paulo, São Paulo, SP Brazil

**Keywords:** Social protection, Social inclusion, Poverty, *Bolsa família*, Food insecurity, Employability, Mortality, Gender roles

## Abstract

**Background:**

The *Bolsa Família* cash transfer Program (BFP) aims to break the poverty cycle by providing a minimum income to poor families conditioned on their investment in human capital (such as, education and health) and currently is the largest Program in the world in terms of the number of beneficiaries. Because there is a scarcity of reviews grouping studies on the impacts of the BFP, the objective of this scoping review was to identify and describe studies which evaluate the impact of the BFP on poverty, health, education, and other related outcomes.

**Methods:**

We searched for quantitative, qualitative, and mixed-method articles that assessed the impact of the BFP on any aspect of the beneficiaries' lives between 2003 and March 2021. We included quantitative articles that used experimental, quasi-experimental or pre and post comparison designs. We excluded articles that analyzed impacts on political outcomes. There was no age restriction for the participants. The search was done in seven electronic databases.

**Results:**

One thousand five hundred forty-six papers were identified and 94 fulfilled the inclusion criteria. Poverty and health outcomes were the most common outcomes studied. We found consistent evidence of the positive impact of the BFP on poverty reduction, as well as employment outcomes. We also found positive impacts in relation to mortality rates for children and adults, school dropout and school attendance among children and adolescents, and violence related outcomes such as homicide, suicide, crime, and hospitalization. However, we also found some evidence that BFP increased intimate partner violence and gender stereotypes among women and no evidence of impact on teenage pregnancy.

**Conclusions:**

Overall, the studies included found that BFP showed positive impacts on most poverty, health and education outcomes. More studies are needed to confirm some results, especially about violence and stereotype against women as there were few evaluations on these outcomes.

**Supplementary Information:**

The online version contains supplementary material available at 10.1186/s12889-024-20046-2.

## Introduction

Poverty has a widespread impact on people's lives, including food insecurity, restricted access to health services, limited access to professional development and marginalization of the sharing of benefits resulting from the economic progress of society [1]. The lack of financial resources, in the case of extreme poverty, reduces access to basic living conditions, such as food quality, housing, and basic health services [2]. There are various measures for assessing the population's level of poverty, many developed as a result of the understanding that poverty is a multidimensional phenomenon. Among these, ‘monetary’ or ‘income’ poverty continues to play a central role in poverty assessment and monitoring [2]. Living in poverty is characterized as having resources far below the population average, which varies depending on the context of each country and the type of poverty measurement used. This condition may result in exclusion from the living conditions that the majority of the population has access, achievement of positions of power and spaces of entertainment of the society in which the person or family live [2, 3].

Since the 1990s, cash transfer programs (CTP) have been widely adopted as a strategy to reduce poverty in low- and middle-income countries aimed at families that are experiencing a shortage of financial resources [4]. The *Bolsa Família* Program (BFP) is the Brazilian CTP created in 2003 by the federal government to break the poverty cycle by providing a minimum income to poor families. The BFP underwent changes in income cut-off amounts for eligibility, as well as in transfer amounts, between 2021 and 2023. In this article, we will discuss the program according to the regulations in place until the end of 2020. The primary characteristics of the program, based on the BFP valid at that time, are presented below.

The Program offers unconditional income transfers to families in extreme poverty (those with monthly income per person of up to about BRL 89.00 or USD 16.05^1^) and conditional transfers to families in poverty (those with monthly income per person between BRL 89.00 or USD 16.05^2^ and BRL 178.00 or USD 32.09^2^) where there is at least one child/adolescent aged between 7–17 enrolled in school, or a pregnant or breastfeeding woman in the household [5, 6]. In more detail, there are four types of benefits provided by the BFP: (i) If the family has a child or adolescent aged 0 to 15 years, the family is entitled to receive BRL 41.00 (or USD 7.39) per month; (ii) If the family includes an adolescent aged 16 or 17 years, they are entitled to receive BRL 48.00 (or USD 8.65); (iii) If the family has a pregnant woman, they are entitled to receive BRL 41.00 (or USD 07.39) per month for a period of nine months; (iv) If the family includes a child aged between zero and six months, they are entitled to receive BRL 41.00 (or USD 07.39) per month. Educational conditionalities are adolescents aged 16 or 17 years must be enrolled in school and maintain a minimum attendance rate of 75%; children and adolescents aged six to 15 years must be enrolled in school and maintain a minimum attendance rate of 85%. Health conditionalities include that children up to seven years old must receive the recommended vaccinations from health teams, be weighed and measured, and undergo growth and development monitoring; children aged between zero and six months must receive the recommended vaccinations from the health team and be weighed; breastfeeding or pregnant women must obtain pre- and postnatal health services [5, 6].

The BFP has been well established for decades and currently reaches 14 million families (20–25% of the total population). Different from other cash transfer programs, such as *Oportunidades* in Mexico, no controlled evaluation of BFP was implemented at its inception. Even today, the impacts of BFP in the lives of their beneficiaries lacks a periodic systematic assessment, which is crucial for stakeholders to identify aspects needing changes and those yielding the desired effects. This assessment could enhance governance, cost-effectiveness, and the impacts of implementation. In recent years, however, with the objective of reducing the BFP's deficiency in assessments of this nature, the Ministry of Social Development created a task force to analyze whether modifications to the program, such as adjustments to the monthly fee, have produced tangible effects on various aspects, including hospital admissions and school retention.^2^ At the same time, several studies have been published investigating the outcomes of BFP [7]. To date, there is a scarcity of reviews in literature that synthesize these findings, integrating various outcomes. Summarizing the potentially broad impacts of BFP could inform public policies aimed at alleviating poverty. The single scoping review available analyzed scientific literature published between 2003 and 2020, associated with documentary research on government websites, with the objective of identifying the contributions of the BFP to the reduction of income inequities, improvements in food and nutrition outcomes, and enhanced access to basic social rights in the areas of education and social assistance. Their review is restricted to these outcomes, as the articles were grouped by specific thematic areas previously defined, which did not include, for example, articles that evaluated impacts of BFP on gender and race inequities, reducing violence or promoting autonomy. Neves’ review selected all types of quantitative articles including those without a comparison group and did not include qualitative ones [7].

The objective of this study was to conduct a scoping review of the literature to summarize and describe the reported impacts of the BFP on poverty, health, education, and other related areas of its beneficiaries’ lives, through quantitative research [8]. We build on the previous work by also including qualitative studies which might allow more for exploring mechanisms and capturing the experience of beneficiaries.

## Methods

Scoping reviews are designed to address broad topics where many different study designs might be applied. When the research topic is complex and heterogeneous in nature or has not yet been extensively addressed, it is useful to conduct a scoping review, to describe the literature in this topic in terms of volume, types of evidence and existing gaps [9, 10]. To understand the scientific evidence on the impacts of the BFP on the lives of beneficiary’s individuals, families, and populations, we chose to carry out a scoping review. This scoping review is registered at Open Science Framework (OSF, [11]), where the supplementary material can be found.

### Literature search strategy

We identified studies by searching the following electronic databases: Education Resources Information Center (ERIC), Embase, National Health Economics Information Portal (ECOS), PubMed, Science Direct, and Scientific Electronic Library Online (SciELO). The search was limited to papers published from 2003 (when the BFP started) to March 2021, and it was conducted between 2020 and 2022. We chose to include articles published up to March 2021 because, in April 2021, the BFP was renamed *Auxílio Brasil*, a new version of the *Bolsa Família Program* in response to the COVID-19 pandemic.

The *Auxílio Brasil* increased the benefit amount, establishing for the first time a minimum value of BRL 400.00 (or USD 72.12^3^) that each participating family was entitled to receive. This change in the program was a positive step, but it did not fully address the country’s demand, as it primarily served to address the backlog of families that were on the waiting list [12]. Other changes included the suspension of the conditionalities, which would be reinstated once services resumed their traditional operations, and the increase in the value of the variable installments. In 2023, the BFP was reinstated, now providing a minimum of BRL 600.00 (or USD 108.17) per family, and an additional BRL 150.00 (or USD 27.04) per child up to six years of age.^4^

The search strategy was customized for each database. The terms were related with cash transfers (those we selected from a systematic review with metanalysis already published by our group – 13) and the term ‘Brazil’. A list of all the search terms and how these terms were combined can be found in the supplementary chart 1.

### Inclusion and exclusion criteria

#### Inclusion

There was no restriction on the language of the papers. We included quantitative, qualitative, and mixed methods research articles, which must be peer-reviewed scientific papers.

Inclusion criteria for quantitative papers, using PICO: (i) population: BFP beneficiaries, including families or populations, regardless of age and gender; (ii) intervention: the BFP; (iii) comparison: we included cross-sectional, longitudinal panel data, cohort studies or articles that used experimental or quasi-experimental methods to estimate the effect of BFP, as for example including a comparison group balanced in sociodemographic characteristics, with the only difference that one group received BFP and other did not; it also included papers with pre and post comparisons, i.e., where they serve as their own control; (iv) outcome: studies that analyzed the impacts of BFP on any outcome, without prior definition by the authors of this review, such as health, poverty, education, employability, gender inequality, fertility, and violence.

Qualitative papers were included because this method brings another perspective of the impacts of the BFP, due to its nature of investigation where in-depth interviews allow us to understand about the impacts of the program on beneficiaries’ daily lives. Furthermore, we could capture other areas that were not explored in quantitative research with robust designs. We included studies with primary or secondary data where there were presented reports from any informant and regarding any outcome on the impact of the program, without prior definition by the authors of this review, such as reports from beneficiaries, health or social professionals, about impacts they perceived of BFP on beneficiaries or health professionals, about impacts they perceived of BFP on beneficiaries’ health, income, education, gender inequality, and other aspects not mentioned in advance, regardless of age and gender.

#### Exclusion

For both quantitative and qualitative studies we excluded the grey literature.

Exclusion criteria for quantitative papers, using PICO: there were no excluded population or intervention; (i) comparison: those who compared the prevalence of some variable between beneficiaries and non-beneficiaries with bivariate analysis; (ii) outcome: papers that only analyzed the impact of BFP on national/municipal elections, because it was beyond of the aims of this review.

Exclusion criteria for qualitative studies: we excluded studies that were focused on evaluating characteristics of the implementation of the program (coverage, perceptions of professionals about conditionalities, etc.) rather than impacts on beneficiaries.

### Screening process and inter-rater reliability

The list of all studies identified were checked to eliminate duplicates using the Mendeley automatic duplicate elimination mechanism, then checking by hand the titles, abstracts and DOIs of the publications to find remaining duplicates. The publications without duplicates were transferred to an Excel spreadsheet.

Two authors (JM, CZ) independently screened the title and abstract, then full text against inclusion and exclusion criteria. When the two reviewers disagreed in the selection, a third or fourth author (CSP, AM) reviewed to achieve consensus.

The initial agreement between the two reviewers was 86%, after discussion the agreement was 97% and only 3% were resolved by CSP and AM.

### Data extraction

For included studies, regardless of whether they are quantitative, qualitative, or mixed methods, data were extracted on: (i) study design; (ii) geographic location where the study took place; (iii) outcome; (iv) sociodemographic characteristics of the sample; (v) methods of recruiting participants; (vi) how were the outcomes assessed; (vii) who completed the assessment; (viii) main results. Data extraction was conducted independently—articles were divided, and each was reviewed by a single reviewer for data extraction (JM or CZ).

In the quantitative studies, the themes/outcomes were extracted from the objectives or in the definition of variables in the statistical analysis of the original manuscripts. In contrast, for the qualitative studies, to identify and define themes, we relied on keywords present in the informants' discourse, such as 'school attendance,' 'acquisition of material goods,' 'poverty alleviation,' and 'job seeking.' Additionally, it was important for us to interpret generally what the accounts referred to; however, none of the strategies used in qualitative studies were carried out using any discourse interpretation techniques.

For the quantitative studies, to classify the outcomes as positive, negative, or neutral, we relied on the statements of results as reported in the original studies. Terms such as "positive impact" and "negative impact" were predominantly used. In their absence, we referred to terms like "increase" or "decrease" to denote significantly positive or detrimental aspects of the outcomes. For example, an article reported "increase in school attendance rates among children" and then, we considered this to be a positive outcome. Specifically, in the qualitative studies or in the qualitative part of the mixed-methods studies, the results were described based on the respondents’ point of view, their statements that referred to the contribution, impact, change or effect of the BFP on a specific outcome, which were important to understand the context and meanings of the Program for them. We used the qualitative results to complement the quantitative findings, which were our primary focus in the present review. They were used to illustrate participants' perspectives and to help interpret the more objective results. Qualitative papers provide another view of the impacts of the BFP due to their nature of investigation. In-depth interviews allow us to understand the experiences of the beneficiaries, but these studies usually do not aim to test impact. Furthermore, qualitative research enables us to capture areas not explored in quantitative studies with robust designs.

Firstly, we assessed whether an article has positive, negative, or neutral results for each of the outcomes it analyzed. Thus, at the level of articles, the same article may have a positive result in education and a negative result in nutrition, for example. A second level was based on each of the outcomes considering the results of all papers together; this was used to reach a conclusion on every specific theme/outcome. For example, if most articles showed positive results of the BFP on reducing mortality, we define this as general positive impact on this subtheme and mentioned the small percentage of negative results (same rule when most results were negative). We inform the percentage of articles that showed positive, negative, and neutral results into each theme. The overall conclusion of all outcomes inside of a theme (subthemes, e. g. social inclusion, infectious diseases, and violence against woman) will be summarized as result of the Table 1. The results in terms of broad outcomes (e. g. poverty, health, and gender equality) are presented in the conclusions of each line of Table 1.

**Table 1:** Description of the quantitative papers and quantitative results of mixed papers, on the impact of the BFP according with the seven themes and subthemes (*N* = 64) (the number within the table represent the study's reference)

Themes	Age range of participants	Sample data source		Results	Conclusions
		**Primary data**	**Secondary data**		
**Poverty (*n* = 25)**					
*Food consumption (n* = *7)*	Adults	-	14^a^, 30	One study found that the consumption of food from families beneficiaries of the BFP was higher than among families not benefited by the Program (30). Studies found that BFP provided the diversification of food from the basic basket (2, 47), and improved the probability of consumption of foods derived from milk (14). One study showed that before entering the BFP, 50.3% of the participants faced severe food insecurity, and this number went down to 36.8% in five years (9) One study indicated that the BFP had a neutral impact to the consumption of healthier foods (2). Only one study showed that BFP investment was associated with increased child malnutrition amongst the poorest subsample of municipality’s population (31)	Five studies indicated an improvement in the quantity and quality of food intake, and one of them showed no improvement (neutral) on the consumption of healthy food. One study showed that BFP increased child malnutrition
	All ages	9^cd^	47		
	NP^b^	-	2, 31^c^		
*Macroeconomic variables and household income (n* = *9)*	School age	-	15^c^	Studies indicated that the expansion of the BFP was associated with the reduction of economic inequality (1, 15) and changes in macroeconomic variables (28), with greater impact on non-metropolitan and poorly industrialized municipalities (77). One study found that BFP increased protein production across Brazil: and its rates tend to be greater in north-eastern states (31). A study showed that Brazil has made progress toward Millennium Development Goals: (i) evidenced satisfactory performance and contribution of the Program to reduce poverty and promote human development; (ii) Beneficiaries’ income increased in 2006, 2009 and 2012 (11). There was a positive effect of BFP on the reduction in a poverty indicator (Foster index) for the Brazilian states (7). Similarly, a study with national data from the IBGE indicated the contribution of the BFP to break the “cycle of poverty” (65). BFP was positively associated with increase in wages and complementary incomes (21). However, a study showed that the Program did not produce statistically significant effects on income inequality rates (7)	Eight out of the nine studies demonstrated positive impacts either on income of families or macroeconomic variables. Otherwise, one study did not identify (neutral) impacts on income inequality rates
	All ages	-	11^c^, 77		
	NP^b^	-	1, 7, 21^c^, 28, 31^c^, 65		
*Social inclusion (n* = *5)*	Adults	63, 79^d^, 92^d^	76	Three studies have identified a positive impact of the BFP on the purchase of products, such as consumption of goods and services of a private nature (63), housing (76) and expansion of inclusion in socialization spaces, such as schools, health centers and commerce (79). One study showed that the BFP contributed to the autonomy of beneficiaries, allowing to overcome important forms of social deprivation (92). One study identified that a small proportion of adult participants were able to return to school and increase their educational qualifications (neutral) (9)	Four studies showed positive impacts of BFP on the social inclusion of beneficiaries. One study identified neutral effect
	All ages	9^cd^	-		
*Food quality (n* = *4)*	School age	-	89	One study identified that the BFP increased the intake of energy and macronutrients and decreased the intake of calcium and vitamin A, D, E and C of beneficiary adolescents. Adult beneficiaries from the Southeast increased the intake of fibers, iron and selenium, and those in the Northeast decreased the intake of energy, lipids, among others (88). One study identified that BFP was effective in increasing the quality of the diet of families, mainly improving the variety and reducing the consumption of fat and sodium (20). It was found that the proportion of children and adolescents with low weight was lower in beneficiary families than in non-beneficiary families in the Northeast region (89). It was also found that BFP beneficiaries had lower consumption of processed and ultra-processed foods compared to non-beneficiaries (90)	All four studies showed a positive impact of the BFP on the quality of feeding of children and young beneficiaries
	All ages	-	88, 90		
	NP^b^	20	-		
**Health (*n* = 23)**					
*Utilization of health services (n* = *1)*	All ages	9^cd^		This single study showed that after receiving BFP, families have been able to access healthcare services on a more regular basis. Particularly women who were systematically excluded – black women, poorly educated and from the less developed regions of the country –, after their participation in the BFP, increased access to prenatal care and could count with a greater availability of public healthcare network (9)	Positive changes were observed in healthcare access, particularly amongst vulnerable population
*Child health (n* = *4)*	Preschoolers	81	6, 39, 85	One study found that the BFP increased the chances of children visiting the health center and improved their behavior and social relationships (81). Two articles demonstrated that the BFP did not affect the immunization rate of children (6, 85). One study found that the Program was associated with a reduction in the z-score of length and weight for age of beneficiaries (39)	The BFP increased the chances of children visiting the health center, as well as their psychosocial health (one article), and two articles demonstrated neutral effects of BFP on immunization rate. One paper showed that BFP did not improve the immunization rate. There was a negative impact of reduction in children growth (one article)
*Infectious diseases (n* = *8)*	School age	-	25, 58	One study found that the incidence of tuberculosis decreased in municipalities with high BFP coverage (57), and three studies showed that the BFP had a positive impact on the cure rates of tuberculosis patients (12, 60, 73). Three studies identified a reduction in the rates of new leprosy cases (25, 58, 66), and a fourth study identified increased treatment and cure of this disease (67)	The eight articles pointed out positive impacts in the fight against tuberculosis and leprosy
	Adults	73, 60	-		
	All ages	-	67		
	NP^b^	-	12, 57, 66		
*Mortality (n* = *10)*	Preschoolers	-	11^c^, 31^c^, 35, 72, 82, 83, 84	One study showed that BFP spending mitigated harmful health effects, especially among vulnerable populations (37). Another study showed that a greater reduction in the risk of death occurred among those receiving the BFP benefit compared to those who did not receive (38). It was found that the increase in BFP coverage resulted in a reduction in the mortality rate due to tuberculosis (29). Six studies found that the mortality rate of children under 5 years of age decreased as BFP coverage increased (11, 35, 72, 82, 83, 84). One study showed that BFP was associated with increased infant mortality (31)	Nine articles indicated positive effects on mortality rates in children and adults, in contrast with only one study
	Adults	-	37, 38		
	NP^b^	-	29		
**Education (*n* = 12)**					
*School enrolment, approval and dropout (n* = *5)*	School age	27	5, 10, 34, 86	It was found that the BFP contributed to reduce the chances of school dropout and increased rates of year approval (5, 10), and one study identified this impact on the sample of girls living in rural areas, with the greatest effect on adolescents (15 to 17 years old versus 6 to 14 years old) (27). A study showed that at grades 1–8, the Program provided an increase in the number of students enrolled, lowered dropout rates, and raised grade promotion rates (34). One study identified a significant and negative impact of BFP in performance of schools in Portuguese proficiency by Prova Brasil between 2005 to 2007 and in Mathematics for the year 2007 (86)	Four studies indicated contribution to permanence among children and adolescents, but one study identified a negative impact of BFP in Mathematics and Portuguese performance
*School attendance (n* = *7)*	School age	31^c^, 50	8^c^, 15^c^, 19^c^, 56, 74	Four studies showed that children and adolescents receiving the BFP had a higher school attendance compared to those who did not receive the benefit (15, 50, 56, 74), also when this estimative were compared amongst a same group of adolescents before and after receiving BFP (19). It was found that BFP is positively associated with primary and secondary school attendance (31); and that there were positive and significant effects on girls’ school attendance (8)	All the studies showed a positive impact on the school attendance of children and adolescents
**Employability (*n* = 10)**					
*Participation in the labor market (n* = *7)*	All ages	9^cd^	19^c^	Four studies showed a statistically significant association between the receipt of the BFP and the increase in participation in the labor market (9, 21, 91, 94). It was found a growth in the proportion of beneficiaries with formal jobs, which went from 9.8% in 2004 to 12.5% in 2007 (9) and influence on the choice of young people to study and work at the same time (19). Otherwise, two studies showed that BFP did not increase labor force participation among adults (8, 19)	Five studies indicated that the BFP was associated with increased participation of young people and adults in the labor market, in contrast to two other studies that indicated neutrality
	Adults	-	8^c^, 94		
	NP^b^	-	21^c^, 91		
*Working hours (n* = *3)*	All ages	-	19^c^	A study identified that the working hours of the beneficiaries decreased, allowing better quality at work (22). One study showed that poor heads of households who have land tenure had an increase in their chances of executing agricultural work (59). It was found that BFP did not increase the number of working hours among mothers and fathers (19)	Two studies showed that the BFP positively influenced the quality of work. One study showed neutral effect
	Adults	-	59		
	NP^b^	-	22		
**Gender equality (*n* = 4)**					
*Gender roles, stereotypes (n* = *1)*	NP^b^	-	62	One study identified that the BFP increased domestic care time for women and reduction for men (62)	This study indicated the reinforcement of traditional gender roles
*Violence against woman (n* = *2)*	Adults	41^cd^	51	A study with data extracted from the 2009 National Household Sample Survey indicated that the BFP increased domestic violence (51). Another study identified that the BFP had no influence on feminicide rates (41)	BFP did not have effect on feminicide and can increase domestic violence
*Women’s empowerment (n* = *1)*	Adults	41^cd^	-	One article showed that BFP was associated with an increase in separations, and, to a greater extent, separations of couples with children (41)	BFP increased divorce
**Teenage pregnancy (*n* = 3)**					
*Pregnancy rate (n* = *3)*	Adults	-	17, 61, 87	One study identified that the BFP reduced teen pregnancy rates among poor women living in urban areas, bringing them closer to the Brazilian average (61), and other study showed that teenage pregnancy rates among BFP beneficiaries were significantly lower than those of non-beneficiary adolescents (87). Otherwise, one study showed that beneficiaries of BFP were more likely to generate the second child compared to non-beneficiaries (17)	Two of the three studies indicated an impact on reducing teenage pregnancy, while the third indicated an increase in fertility
**Violence (*n* = 3)**					
*Homicide and other crimes (n* = *2)*	School age	-	18	Studies identified the Program’s impact on reducing crime rates (18) and in the rates of hospitalizations for violence (not specified what type) (42). It was found that the rates of homicide were negatively associated with the duration of BFP coverage (42)	BFP reduced crime, hospitalizations for violence rates, and homicide
	NP^b^	-	42		
*Suicide (n* = *1)*	NP^b^	-	4	One study found that as BFP coverage increased, suicide rates decreased in several municipalities in different regions of the country (4)	The BFP led to a decrease in suicide rates

^a^All the references are presented on the supplementary chart 2; NP^b^ = not presented; ^c^ These studies are in more than one category and/or subcategory; ^d^ Mixed methods study

To identify which articles had the multi-theme, there is the symbol “§” next to the article’s reference number. Some articles were also divided into two tables: the first has quantitative articles and quantitative results from mixed method articles (Table 1); and the second has qualitative articles and qualitative results of mixed method articles (Table 2). Therefore, papers with mixed method will appear in both tables (Table 1 and Table 2) and, in order to identify which are them the symbol “**” was added.

**Table 2:** Description of the qualitative papers on the impacts of the BFP according with the seven themes and subthemes (*N* = 50) (the number within the table represent the study's reference)

Themes	Age range of participants	Sample data source			Results	Conclusions
		**Primary data**		**Secondary data**		
**Poverty (*n* = 20)**						
*Macroeconomic variables and household income (n* = *9)*	Adults	3^c^, 13^c^, 36^cd^, 64, 69, 80		-	People form a riverside population in the Amazon stated that the BFP helped in the economic organization of families and provided a greater sense of stability (80). A study with beneficiaries of the city of Campinas (São Paulo State) revealed that beneficiaries stated the relevance of the Program as “help” and “complement” in income (69), while for beneficiaries of Carnaubal (Rio Grande do Norte State), and a riverside community of São Carlos (Rondônia State) the BFP assisted in the acquisition of food (36, 40) and the permanence of people in the field/cultivation of land (40). Beneficiaries pointed that the Program was a regular income opportunity, able to minimize the effects of seasonality, expressed in temporary jobs, mainly in agricultural activities (32). According to beneficiaries’ speeches, this family per capita income is essential for them to have no concern with the quantity or quality of their families’ diet and their own (9). According to unit health professionals, the BFP reduced poverty amongst beneficiaries (13). Beneficiary mothers stated that the Program gave them the possibility to offer their children consumption of goods that they did not have the opportunity to acquire throughout their lives (64). Also, beneficiaries affirmed that BFP had positive effects on living conditions through the use of resources to meet: (a) basic needs; (b) payment of water and electricity bills; (c) payment in installments for durable goods; and (d) payment of language courses and professional qualification. Otherwise, it can stimulate household indebtedness (3)	In all nine studies, beneficiaries said that BFP had positive impact on family income in different populations
	All ages	9^cd^		-		
	NP^b^	32^c^, 40^c^		-		
*Social inclusion (n* = *9)*	Adults	3^c^, 36^cd^, 75		53^c^	In four different studies, BFP beneficiaries stated a positive impact of the Program on the purchase of products, such as school supplies, clothing, and shoes (36), consumption of goods and services of a private nature (24, 53, 75), and expansion of inclusion in socialization spaces, such as schools, health centers and commerce (32). Beneficiaries reported that they had better access to schools, formal markets and transport networks compared to the period prior to receipt (33). According to them, the Program allowed access to universal services, which generated a perception of improvement in the self-esteem and happiness of the beneficiaries. However, respondents felt stigmatized for being beneficiaries of a cash transfer policy (46). Beneficiaries said that BFP contributed to their autonomy, allowing to overcome important forms of social deprivation (48). They also said that BFP improved their self-assessment as a consumer, as a bank card holder and/or as the one who was able to pay his bills on time (3)	Beneficiaries felt that the BFP positively impacted their social inclusion, according to all the nine articles
	NP^b^	24^c^, 32^c^, 33, 46, 48		-		
*Food quality (n* = *1)*	Adults	93		-	In a study with 38 beneficiaries of Curitiba Municipality, participants reported low variability in food intake (93)	Beneficiaries reported a negative impact of the BFP on the variability of food intake
*Food insecurity (n* = *1)*	Adults	55		-	Beneficiary families declared a positive impact on access to food, with improvements in their well-being, standard/quality of life and on the development of credit practices (finance the consumption), important aspects to combat food insecurity (55)	Beneficiaries felt that BFP contributed to the reduction of food insecurity
**Gender equality (*n* = 18)**						
*Gender roles, stereotypes (n* = *4)*	Adults	-		53^c^	Women beneficiaries declared that the BFP tended to reinforce traditional gender roles (32, 53), such as the greater presence of women in the domestic sphere (24). Women beneficiaries and non-beneficiaries of the BFP attributed moral judgments to the different beneficiary families, such as the perception that “new mothers have less legitimacy than the others” and that “if the family has many assets, it means that the family does not configure itself as ‘poor’ and, therefore, does not deserve the aid” (45)	Beneficiary women declared stereotypes and/or negative moral judgments/pre-judgments in relation to them
	All ages	45		-		
	NP^b^	24^c^, 32^c^		-		
*Violence against women (n* = *3)*	Adults	3^c^, 41^cd^			The qualitative component of one mixed method study with 13 people indicated that the BFP had heterogeneous responses about impacts of BFP on violence, such as it can increase, decrease, or have no influence on intimate partner violence (IPV) (41). A second study with 31 beneficiaries and BFP`s managers showed that BFP makes possible to promote improvements in domestic situations of embarrassment, harassment, and violence against women (3). A study with data extracted from the 2009 National Household Sample Survey indicated that the BFP tended to increase violence against women (51)	Beneficiaries perceived the possibility of promoting positive changes in family atmosphere, according to one study. The results about impacts on violence against women were heterogeneous
	NP^b^	-		51		
*Women’s empowerment (n* = *11)*	Adults	3^c^, 16, 23, 52, 54^c^		-	Women beneficiaries of the BFP reported that the benefit contributed to greater financial autonomy and improvement of empowerment (3, 16, 23, 40, 52) and social recognition and dignity (78). One article showed that BFP had meaningful impacts on women’s control over decision making, but with considerable heterogeneity (26). Beneficiaries reported that BFP helped minimizing the vulnerability and deprivation of women, which did not have to submit to jobs often offered almost free of charge on the job market (32) Beneficiary women affirmed that they did not feel discouraged from seeking employment, which challenges the idea of a possible “laziness effect” (54). When questioned (the beneficiaries) about the means to achieve a dignified life, the most cited set was “education-work-money” (43). A study on racial issues pointed out that the BFP exerted more influence on the daily lives of black women compared to white women, however, without impact of equalizing the situation between black and white women (44)	The BFP tends to contribute to improve the self-esteem of women beneficiaries, as well as to their greater financial autonomy, despite heterogeneity in the results
	NP^b^	32^c^, 40^c^, 43, 44		26, 78		
**Education (*n* = 7)**						
*School enrolment, approval and dropout (n* = *5)*	School age	-		34	Mothers said that children and adolescent beneficiaries of BFP remained in school at the expense of working in the field, allowing them to continue their studies (70). Mothers report that because of BFP, the incentive to study prevails to the detriment of work (24). Also, the Program provided an increase in the number of students enrolled (34). Based on the beneficiaries’ speeches, a study showed that young participants and those with a certain level of education see fewer costs and greater benefits in returning to school than older beneficiary people (9). Beneficiaries said that BFP enhanced their enrollment in free courses, such as: computer course, caregivers for the elderly and children, and industrial sewing (32)	Beneficiaries felt that BFP contributed to permanence of children and adolescents in school. Young beneficiaries and those with a certain level of education see fewer costs and greater benefits in returning to school than older people
	Adults	70		-		
	All ages	9^cd^		-		
	NP^b^	24^c^, 32^c^		-		
*School attendance (n* = *2)*	School age	13^c^		-	According to primary care professionals, the BFP increased school attendance of children (13). Beneficiaries said that the BFP, besides having practical effects on their children’s school attendance and schooling, strengthens feelings of belonging and social recognition on the part of the beneficiaries, generated by the fulfillment of education requirements (68)	Beneficiary mothers and primary care professionals said that there was a positive impact of BFP on the school attendance of children and adolescents
	NP^b^	68				
**Employability (*n* = 3)**						
*Participation in the labor market (n* = *3)*	Adults	3^c^, 49, 54^c^		-	Women said that obtaining permanent income from the BFP increases the possibility of rejecting occupations in informal, underpaid, and devalued jobs, with repercussions on the prospect of more valued jobs (54). Similarly, other beneficiaries said that BFP ensures the possibility of subsistence of poor families excluded from protected work, previously dependent on donations and/or favors, or whose subsistence implied submission to working conditions considered unworthy and/or of very low remuneration (3). Otherwise, in other study, beneficiaries did not identified changes regarding the insertion in the labor market after inclusion in the Program (49)	Two studies showed that according to beneficiaries, BFP was associated with increased participation of adults in the labor market, while in a third study, users pointed no changes
**Health (*n* = 1)**						
*Utilization of health services (n* = *1)*	Adults		13^c^	-	One study showed that the BFP favored the creation and/or strengthening of ties between users and health professionals and increased the frequency of families in the Family Health Units, but professionals reported difficulties in monitoring conditionalities (13)	Positive changes were observed in the relationship between families and services, but monitoring seems to be a challenge
**Teenage pregnancy (*n* = 1)**						
*Access to contraceptive methods (n* = *1)*	Adults		71	-	In a study with five female beneficiaries, only two said that they were able to obtain the sterilization they were seeking, which shows a lack of access to reproductive rights (71)	In general, beneficiaries said that the BFP did not contribute to access to women's reproductive rights

All the references are presented on the supplementary chart 2; NP^b^ = not presented; ^c^ These studies are in more than one category and/or subcategory; ^d^ Mixed methods study

Data extracted and methods of all articles included in our scoping review can be seen in the supplementary Table 1.

## Results

**Fig. 1:**
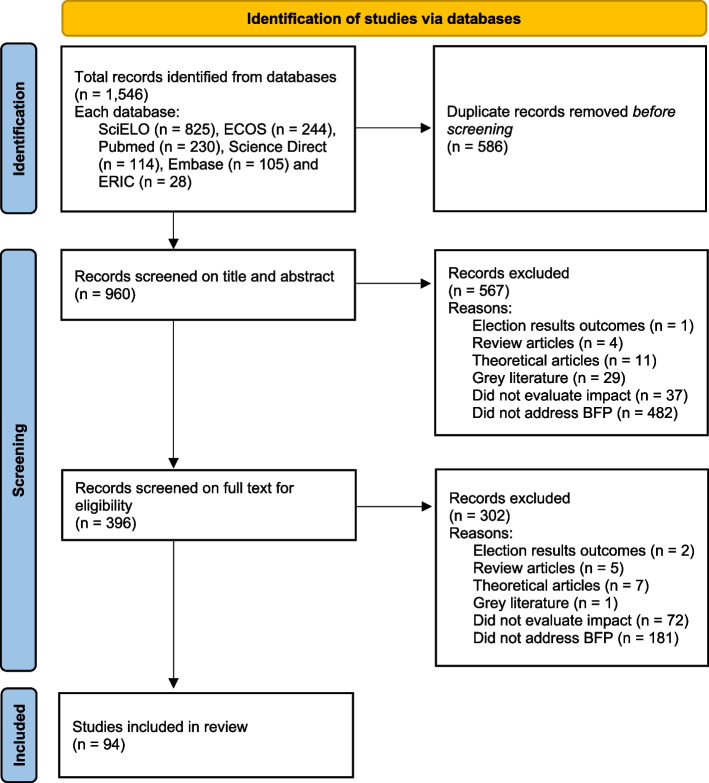
PRISMA flow diagram of selection process of scientific papers

The search of the databases yielded 1,546 publications. After eliminating duplicates, 960 papers remained. After reviewing titles and abstracts, 564 publications (58.7%) were excluded for not meeting eligibility criteria, and after reading the full text, 302 (31,4%) were excluded for not meeting the eligibility criteria too, leading to 94 included articles eligible for data extraction (Fig. 1).

Among the 94 articles, 60 were quantitative, 29 qualitative and 5 were mixed method. For more details on methods of the 94 papers please see supplementary Table 1. We identified a wide variety of outcomes impacting children, adults, and family beneficiaries of the BFP which we classified into the following seven themes: poverty, health, gender equality, education, employability, violence, and teenage pregnancy. Several articles addressed more than one outcome, which means that the same article may be repeated in more than one theme. Frequency of outcomes were poverty (*n* = 36; 33.8%), health (*n* = 23; 21.6%), education (*n* = 15; 14.1%), gender equality (*n* = 14; 13.2%), employment (*n* = 10; 9.4%), violence (*n* = 4; 3.8%), teenage pregnancy (*n* = 4; 3.8%). The studies began in 2009, with the highest number of publications reached in 2014, totaling 17 articles. Since then, the annual number of articles has stabilized between 13 and 14.

### The impacts of BFP

All studies by theme and subtheme, as well as detailed results of each outcome, can be found in Tables 1 and 2. The references for the 94 articles are listed in supplementary chart 2.

#### Quantitative studies (Table 1)

Table 1 describes the 64 articles which performed a quantitative evaluation (4 from mixed methods studies), presenting the impact of the BFP found by the articles on different areas of the beneficiaries’ lives, according to seven broad themes further organized into subthemes. The column “Sample data source” specifies which articles have secondary data and which have primary data. These numbers do not refer to the quantity of articles or its sample size, but the reference number linked to supplementary chart 2 (not the list of references at the end of the article). On the results column, we presented the main findings of each study, and the conclusion column provides a summary of all findings within the same theme and subtheme. Simultaneously, the description of table (core text) is a summary of the results and conclusions of all studies on the same topic. On Table 1, there are seven articles that evaluated multiple outcomes, which are 9, 31, 15, 11, 21, 8, 19.

##### Poverty

It was the most frequent theme assessed (19 articles which brought 25 results) and this comprised four subthemes: food consumption, food quality, macroeconomic variables & household income, and social inclusion (meaning purchase of products, such as school supplies, clothing, and shoes, as well as consumption of goods and services). Social inclusion is a sub-theme of poverty, as the consumption of goods is related to access to spaces and services. Among the 25 results, 21 (84%) of the results of studies showed positive effects (e. g. [14]), three neutral effects (12%) and a single one (4%) negative effect [15].

##### Health

It was classified into four subthemes – utilization of health services, child health, infectious diseases, and mortality. Among the 21 articles on health, which brought 23 results, 20 (87%) of them found positive impacts of the BFP (e. g. [16]), two (8.7%) identified negative effects [15, 17], and one (4.3%) identified no positive or negative effect (e. g. [18]). Overall, most papers showed that the Program had a positive impact on tuberculosis treatment success rates (e. g. [19]), on the reduction in the rates of new leprosy cases (e. g. de [20]), and on reduction in the general mortality rates [21]. Also, according to these studies, BFP increased the chances of children visiting the health center, as well as improved their psychosocial health, according to one study [22]. As mentioned above, negative effects were found in two studies, one showing that BFP was associated with a reduction in length-for-age z-score and weight-for-age z-score in 24 month old children [17], and the other indicating that BFP was associated with increased infant mortality (using data from the national primary information system from 2004 to 2013) [15].

##### Education

The main subthemes for education were school enrolment, approval & dropout, and school attendance. All 12 articles on education identified at least one positive effect among BFP beneficiaries (e. g. [15]). Seven studies showed a positive impact of BFP on the school attendance (e. g. [3]) and four indicated contributions to the reduction of school dropout among children and adolescents (e. g. [23]). Despite the overall positive impact on educational outcomes, there was also one ecological study using data from *Prova Brasil* – census evaluation of public schools in municipal, state, and federal system – between 2005 to 2007 within 5,483 municipalities showed that BFP which had a consistent negative effect, nationally and across regions on school performance rates in Portuguese and Mathematics, among schools with many beneficiary students. The positive side of this data is that over the years, this negative performance decreased [24].

##### Violence

The theme of violence covered homicide & other crimes, and suicide with three articles and four results, all of them with positive outcomes. According to these studies, the BFP reduced crime rates [25], the percentages of hospitalizations for violence (not specified what type), and rates of homicide [26]; BFP led to a decrease in suicide rates [27].

##### Gender equality

Gender equality covered three main subthemes: gender roles & stereotypes, violence against women, and women’s empowerment. There were three articles that together brought four results on gender equality. Two results were negative for gender equality, reinforcing traditional gender roles in the household [28, 29], and one study identified a neutral impact on female homicides and increased divorce rates [30].

##### Employability

The eight studies on employability examined 10 outcomes, which addressed the participation in the labor market and working hours. Seven evaluations (70%) pointed to positive effects of the BFP (e. g. [31]), and three (30%) pointed to neutral effects (e. g. [32]). Most positive results showed that BFP contributed to increase the quantity of people participating in the labor market (e. g. [33]). One result showed that the financial support from the BFP lead to better quality at work due to reduction of excessive hours of work [34]. Two results showed that BFP did not impact on labor force among adults (e. g. [35]) and one result indicated no changes in working hours [36].

##### Teenage pregnancy

Among the 3 studies on teenage pregnancy, two indicated a drop in teenage pregnancy (e. g. [37]), and one indicated an increase [38].

#### Qualitative studies (Table 2)

##### Poverty

It was the most frequent theme assessed (17 articles with 20 results) and comprised four subthemes: food consumption, food quality, macroeconomic variables & household income, and social inclusion. The BFP is consistently quoted as essential for income, for more access to food and social conditions, as can be seen in the following statements:

“[...] I remember that when I had nothing […], many times I went to buy something [at the grocery], and the owner of the store used to ask me how I was going to pay because I didn't have a guaranteed job (teary eyes). After I started receiving the *Bolsa* [BFP], I never went through that again, because they know that every month we have the money guaranteed.” (woman beneficiary; [39]).

“Right now, just recently I bought this stove with the *Bolsa Família* money [...]; I bought the ceramic tiles for this house. […] Then, after I finished [paying] the tiles of this kitchen – [...] Then I bought that kitchen cabinet there, look at it.” (woman beneficiary; [40]).

##### Gender equality

It was the second most researched theme (17 articles based on women reports with 18 results) and comprised three subthemes: gender roles & stereotypes, violence against woman, and women’s empowerment. The importance of the BFP for women’s empowerment is noticeable, as seen in 44% of gender equality studies, as for example in the following quote:

“For me, *Bolsa Família* is a help! Everything has changed, practically everything. I started receiving it when she [second daughter] was five months old. And I took the initiative to move in just because of that, even without work, because I was receiving the *Bolsa Família*.” (woman beneficiary; [41]).

At the same time, some women mentioned that being the BFP recipient may expose them to domestic violence, as seen in the following quote:

“Interviewer: When you received *Bolsa Família*, why did you choose to keep it a secret from your husband?

BFP beneficiary: Because I had a lot of fear of him. He threatened me… he came with a knife, sharp blades… […] If he discovered, he would be mad because he couldn't dominate me anymore. He taught me to say I can't do it without him. He said, “You're mine, you're not of anybody else, and if you run away, I will kill you.” (woman beneficiary; [30]).

##### Education

The main subthemes under the education topic (total of 7 studies/results) were school enrolment, approval & dropout, and school attendance. Participants from six out of seven articles said that BFP contributed to permanence of children and/or adolescents in school, as exemplified in the following passage:

“The positive point is the capacity to eradicate child labor and reduce school dropouts. Improved in this area of education.” (nurse from a primary care center; [42]).

The seventh study analyzed the extent to which BFP could improve the life chances of extremely poor beneficiaries and verified resistances for adult beneficiaries of BFP to return to school, as seen in the following quote:

“Yeah, I thought about going back to school. But when the person is older, there are so many problems with forgetfulness! […] I really would like to have studied when I was younger [...]. For an older person, that is not possible. Now it is my children’s turn. They have studied.” (beneficiary; [43]).

##### Employability

All the three papers (with three results) focused on participation in the labor market. Two studies [41, 44] showed that according to beneficiaries, the inclusion in the BFP does not make women to drop or slow down their search for work, as seen below:

“I also want a job. It could be anything, being a formal job. I want it because it’s more guaranteed!” (women beneficiary; [41])

All studies showed that according to beneficiaries, BFP ensures the possibility of subsistence of poor families excluded from protected work, as seen below:

“My father lived on a farm [...]. He worked using a hoe. That wasn't enough for the children's food [...]. In my time, my father used to say: today you won't go to school because today we must go to the fields, we must weed because otherwise you'll run out of food. [...] So, I tell them [her son/daughter], if I had what you have today that you can study [...] I would be someone in life today! (women beneficiary; [44])”

In a third study, most beneficiaries said that it is difficult to find a formal job:

“In my case I already have a lot of experience in my area. But the issue is the opportunity. It doesn't only involve schooling. It involves opportunities that companies are not offering [...]. And it has gotten worse lately.” (beneficiary; [32])

##### Health

Only one qualitative study covered the impact of the BFP on health showing that the Program seems to increase the attendance and use of health services by beneficiaries:

“I believe that health has changed. The presence of mothers with children at the health center. They bring the kids more often. Pregnant women do not miss appointments. They are fulfilling their obligations better, so to speak.” (nurse from a primary care unit; [42])

At the same time, the conditionalities seems to be challenging and sometimes stressful:

“There are few people who will be assisted during a day. Sometimes they don’t go because of that. Because they don't like it, they think they don't have to wait, that they must to be seen right away. And it gets overloaded! Many keep waiting and many don't like to wait. They leave.” (community health agent; [42])

##### Teenage pregnancy

As well as on health theme, a single article reported women’s experiences on teenage pregnancy, in which women said that there were no improvements on access of contraceptive methods or sterilization:

“[…] I wanted to have my sterilization done because I did not have a job; she [the doctor] asked how many children my husband had, and if he had authorized the tubal sterilization. I said that he already had three children with another woman but hadn't signed any documents; and that I was having my third child. Then the doctor said: ‘Look, I am sorry, but I cannot do the procedure because your husband must sign due to your age. Because if I do sterilize you and he wants to have another child, he can sues me.’. It was bad, I wanted to do my sterilization, but I could not have it." (women beneficiary; [45]).

### Geographical distribution

Our review aimed to identify and describe studies on the impact of the BFP. In this context, we considered it would be interesting to present the origins of the majority of the scientific evidence reported in the article. Knowing the geographical distribution of the studies allows us to identify which regions or Brazilian states are represented and highlights areas with limited data on the impacts of the BFP, thereby encouraging further research.

Initially, we considered all 94 studies when creating the maps. However, 40 of these were national studies (covering all 27 Brazilian States), which did not serve the purpose of the maps –to illustrate the concentration of data in each state or region. Including national studies would have overlapped with regional studies, without providing additional insights. Of the remaining 54 studies, four lacked precise information of where the study was conducted, preventing their inclusion on the maps. Thus, the maps are based on 50 studies.

**Fig. 2:**
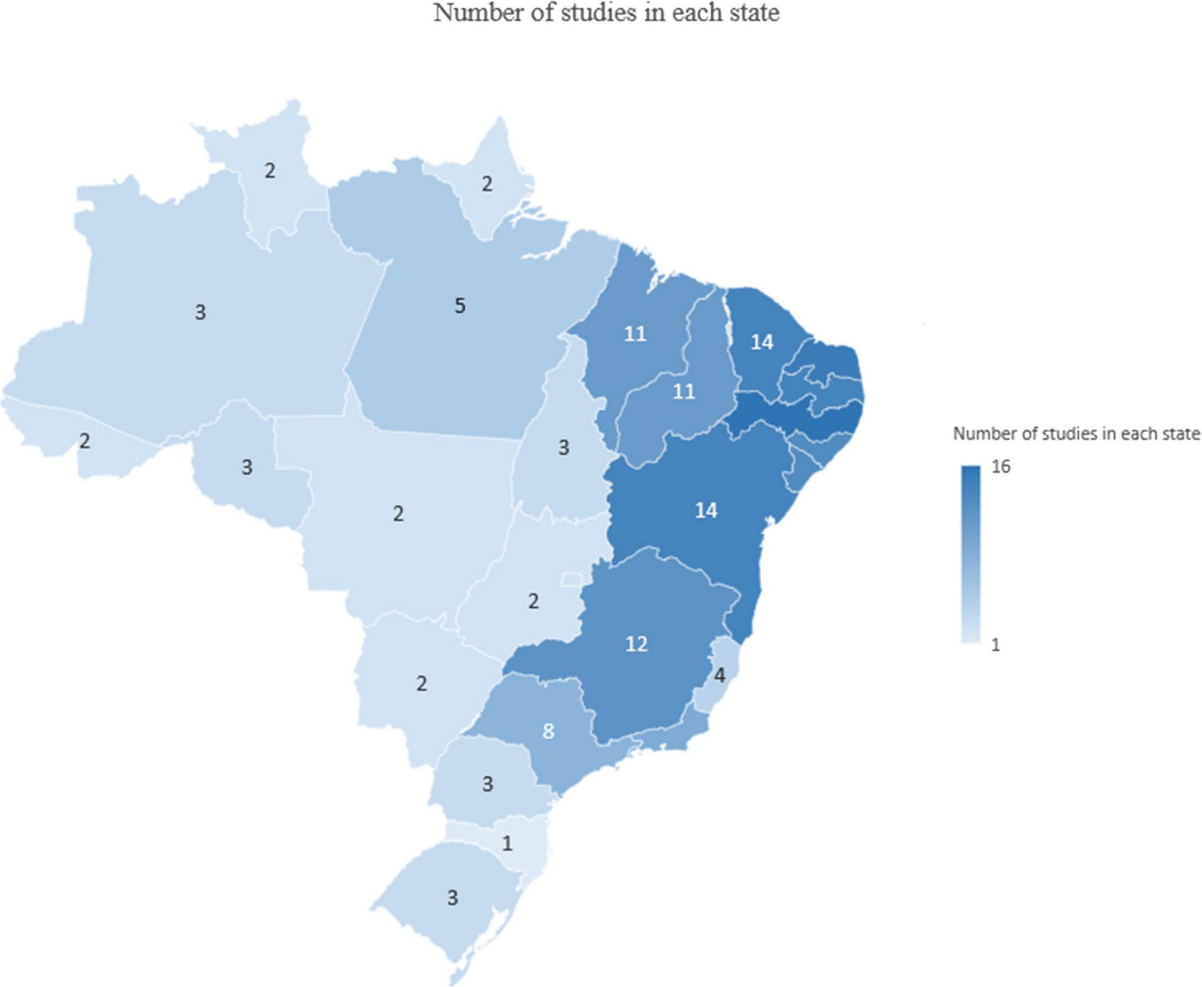
Geographic distribution of studies across states of Brazil (*n* = 50)

The Fig. 2 shows that all states and regions had at least one study, but most were in the Northeast which has the lowest Municipal Human Development Index (HDI) rate (0.663), the highest rates of poverty, extreme poverty, and indicators of income inequality as well as the greatest coverage of *Bolsa Família* beneficiaries in the country [46, 47]. The Southeast which has the highest HDI rate (0.766), according to IPEA Human Development Atlas of 2013 [48] and more human and financial resources had the second most studies [49]. The Midwest had the lowest number of studies.

**Fig. 3:**
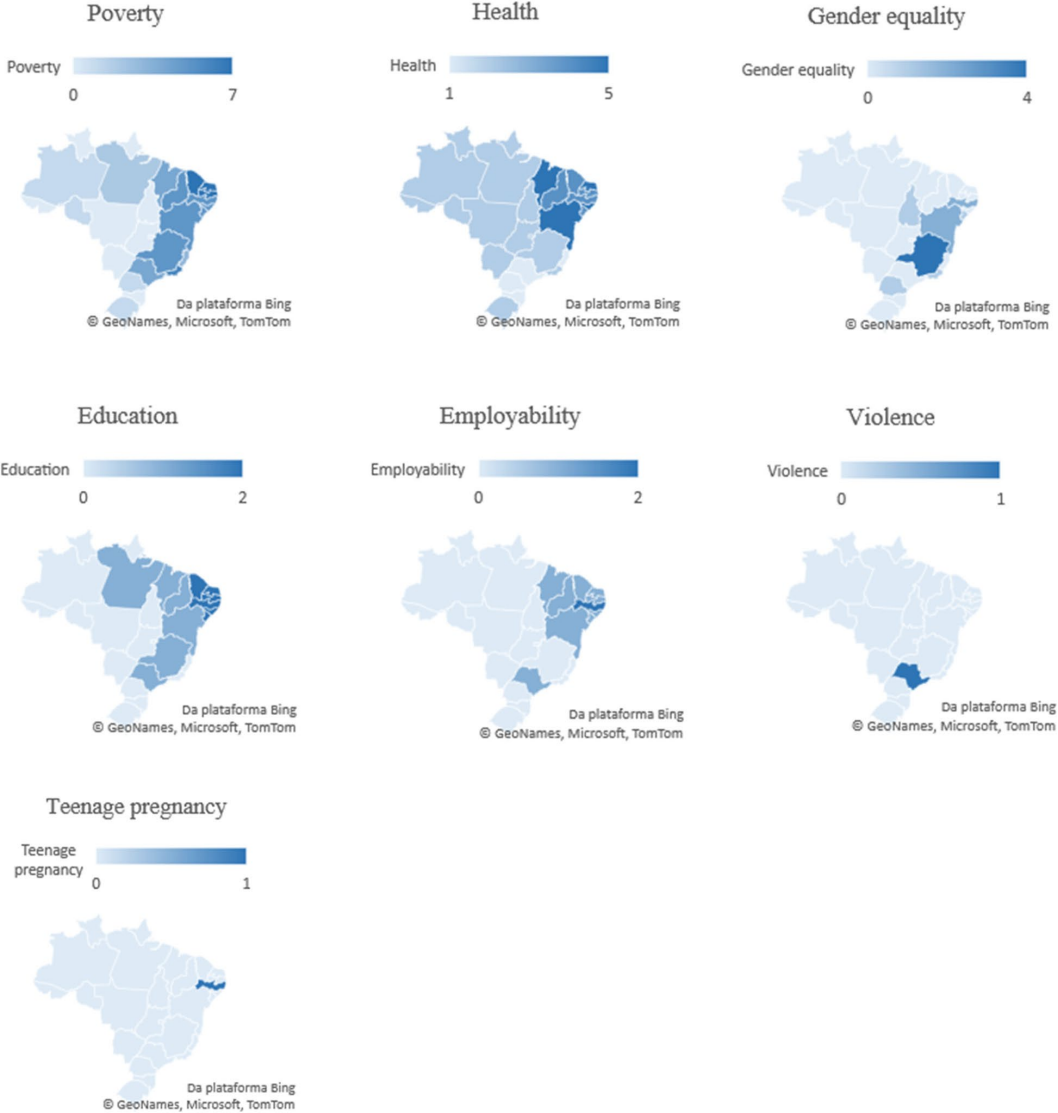
Geographic distribution of studies across states of Brazil, according to the themes (*n* = 50)

The studies on poverty were concentrated in the Northeast and Southeast regions (the poorest ones), studies about health were spread-out all-over Brazil, gender equality and employability studies were focused on the Northeast, while the education theme was spread between Northeast, North and Southeast. Violence and teenage pregnancy were the less frequent topics being concentrated in specific States from Southeast and Northeast regions (Fig. 3).

## Discussion

This scoping review summarized the literature on the impacts of BFP on poverty, health, education, employment, and violence of the beneficiaries, based on 94 articles that met the inclusion criteria. Overall, the literature summarized here indicates that the BFP is generally associated with positive impacts, particularly concerning its conditionalities focused on promoting health and education. Our findings are consistent with the broader literature on the benefits of conditional cash transfer (CCT) programs in low- and middle-income countries [50–52].

Moreover, the BFP consistently demonstrates a significant reduction in poverty levels, which has been widely explored in the literature (e. g. [53, 54]). This addition of money, even if it is small, when the family is very poor, makes a significant difference. It is known that poverty may reduce life chances and the possibility for people to be intentional with their spending, due to the need to invest most of their money in basic needs. It can make it difficult for people to direct their spending towards fulfilling dreams and achieving their goals. With the increase in income conceived by BFP, people can feel control and autonomy towards their decisions, even spend on their objectives, allowing better possibilities of future [55, 56].

This scoping review identified consistent evidence that BFP contributed with health (e.g. [19, 57]). The evidence of enhanced aspects of health can be a result of the conditionality required by the Program, which increases adherence to the health service and consequently contributes to the fight against diseases [58]. On the other hand, it did not improve the immunization rate [18, 47], probably because Brazil had reached a vaccination coverage rate of approximately 100% in 2004 and 2005 [59], covering the period when that study was carried out. Also, according to a single study, BFP had negative effect on growth in height-and wright -age z-score (LAZ) and weight-for-age z-score (WAZ) at 24-month-old children [17]. International literature shows that CCTs can provide an increase in the variety of foods consumed but may be accompanied with an increase in the consumption of high-calorie foods with low nutritional value, which can justify, to some extent, the poor child growth [60, 61].

Evidence regarding the impacts of the BFP on mental health is scarce, limiting conclusions about effectiveness. This scoping review identified one cross-sectional study and one longitudinal study evaluating BFP's impacts on mental health. The cross-sectional study reported improved psychosocial health among children under 7 years of age [22], while the longitudinal study showed a decrease in suicide rates among beneficiaries in general [27]. Studies regarding CCTs worldwide, particularly from Africa and Latin America, has shown that increased income from cash transfers positively influences several key variables, such as individual self-esteem and access to culture, household economic security, and the community environment, which ultimately may improve mental health [4]. The literature on the mental health impacts of various CCTs has grown in recent years, providing robust evidence despite heterogeneous results. A systematic review with meta-analysis [13] screened 12,116 articles and found that, among the 12 articles included, most studies demonstrated a significant positive impact of CCTs on at least one mental health outcome in children and young people. However, none of them reported a positive effect on all the mental health outcomes examined, presenting high heterogeneity. Additionally, the seven studies selected for the meta-analysis showed no impact of CCTs on depressive symptoms. Overall, the recent studies suggest that income transfers alone may not be sufficient to improve mental health. However, when combined with a design that addresses the behavioral and contextual barriers faced by youth, families, and communities, they can be effective even in the long term [4, 62, 63]. A good example is a multicenter study involving three countries in Africa, which found that cash transfers reduced self-perceived stress in Malawi, while programs in Ghana and Tanzania showed no impact on this outcome. The authors conclude that the mixed findings indicate that cash alone may not be sufficient to address the numerous challenges associated with poverty, and that complementary programs may be necessary to improve mental health [64].

BFP had positive effects on education, mainly avoiding dropouts and increasing school attendance (e. g. [65]), but one multinational study showed a negative impact in school performance [24]. Conditionalities in education were more effective at improving school attendance, something to be expected if the conditionalities are about school attendance and no policies are added to improve school performance. That said, we can see that the effect of the BFP is important but limited. A systematic review that evaluated the effects of the BFP on educational indicators among the beneficiaries, pointed out that the Program is not yet able to have a satisfactory impact on the quality of education [66], which would probably need changes in the broader educational system [24]. Thus, even with limitation, BFP meets their goal of increase rates of adolescents staying in school, which is important because it avoids risks of child labor [67], exposition to drugs [68], being victim of violence [25] and teenage pregnancy [37]. There is a certain relationship between more time in school and better life chances, e. g. better jobs [69].

Below we will discuss the outcomes that go beyond the key objectives of the BFP. As expected, there were less studies under the themes that for this review were classified in: employability, gender equality, violence, and teenage pregnancy. It is also noticeable that the evidence is less pronounced and more mixed for those ‘secondary’ outcomes (outcomes not targeted at program’s objectives).

Overall, the studies showed that BFP had positive effects on employability [33, 36]. An important aspect from our review was to find evidence to discuss the ‘common-sense label’ that beneficiaries of BFP would accommodate and not search for jobs, and the results showed the opposite of this ‘common-sense label’. Studies addressing employability indicated that the BFP contributed to an increase in the participation of adults in the labor market. For example, one study with data from all regions of the country showed that BFP allowed young people to choose studying and work at the same time [36], suggesting an absence of the label of “laziness effect of BFP”.

On gender equality BFP had heterogeneous effects [28, 30]. An unfavorable impact was identified with the establishment of stereotypes and/or negative moral judgments/prejudgments in relation to the Program's beneficiaries, particularly about women [69]. Furthermore, two studies demonstrated that BFP could increase violence against women (e. g. [28]). The author [28] points out that the result of an increase in violence among BFP beneficiaries must be taken in caution due its methodological limitations because the results were based on secondary data from PNAD and not designed to test the impact of the BFP. The dilemma between autonomy and the increase in violence against women needs to be further studied because there are few studies in this field.

Results about the impact of the BFP on teenage pregnancy were scarce and controversial: two studies indicate decrease on its rates (e. g. [37]), in contrast to one identifying increase [38]. One eligibility criterion for the BFP is the presence of a pregnant or breastfeeding woman in the family, which brings the debate that BFP could stimulate women to start childbearing earlier or increase pregnancy throughout lifetime, but the current evidence is not enough to prove or to refute either of them. At the same time, it is important to emphasize that there is strong evidence regarding the correlation between increase education and reduced fertility around the world (e. g. [70, 71]), in which the sexual education in schools may play an important help regarding contraceptive methods [72]. Thus, one can expect that a potential long-term effect of the BFP on decrease of teenage pregnancy as consequence of longer school attendance, which is another BFP conditionality.

Interesting, the BFP helped to reduce violence (e. g. [26, 27]). It is known that a large part of the violence comes from the lack of minimum living conditions [26]. Thus, the transfer of income could contribute to the reduction of the crime rate [25, 73].

The qualitative studies of our scoping review allowed us to know some perceptions and relationships of beneficiaries and healthcare workers with the program, which would not have been possible through quantitative research alone. Overall, the studies indicated that beneficiaries and healthcare workers reported perceiving the BFP's ability to mitigate the adverse effects of poverty and improve educational and employment outcomes. However, the studies revealed ambiguous findings in relation to gender equality, which adds complexity to the understanding of this issue. Furthermore, there were legitimate concerns raised about the possibility of an increase in domestic violence, further exacerbating the challenges faced in addressing these intertwined social issues. A recent systematic review of qualitative studies conducted worldwide examined the experiences and perceptions of recipients regarding conditional and unconditional cash transfer programs. The review also showed mixed views, with participants expressing positive and negative experiences [74]. Study by Baranov et al. [75] combined theoretical underpinnings with a meta-analysis to investigate the literature on the effects of cash transfers on intimate partner violence (IPV). The empirical studies encompassed several countries, including Bangladesh, Colombia, Ecuador, and Mexico, and demonstrated that, on average, cash transfers can reduce or have no effect on IPV. Few studies reported an increase in IPV, specifically in physical and emotional violence for women with low levels of education whose husbands with even lower educational levels. According to a theory by Farmer [76], violence may enter into the bargaining process regarding the couple's decisions when the aggressor considers the benefits of using violence against the potential cost of the victim abandoning the relationship. The greater an individual's options outside of marriage, the stronger their bargaining power within the relationship.

In summary, most quantitative and qualitative data mapped by our scoping review showed positive impacts of BFP, mainly on minimizing poverty, promoting health, increasing school attendance, quality of employment and decreasing homicide and crime rates. While negative effects of the BFP were infrequent, our review highlighted the emergence of gender equality as an apprehension, calling attention a concern that should be addressed in future improvements of the BFP.

Describing the results of quantitative studies with qualitative studies reveals several noteworthy points (in this paragraph, to cite the results, we will use references following the order of the supplementary chart 2). Overall, quantitative research on the topic of poverty has indicated improvements in both the quantity and quality of food (e. g. [9]) and an increase in family income (e. g. [30]), whereas qualitative research emphasized reports of enhanced social inclusion, which was largely impacted by the rise in income (e. g. [24]). In the topic of health, overall quantitative results indicated reductions in rates of tuberculosis (e. g. [12]), leprosy (e. g. [25]), and infant mortality [29], while a qualitative study highlighted that families began to use health services more frequently, fostering a stronger bond between families and health service workers [13]. In the education topic, for example, quantitative studies showed that BFP increased school attendance rates among children (e. g. [15]), while qualitative studies revealed that, in addition to increasing school attendance, the program also enhances beneficiaries' sense of belonging and social recognition because they were able to meet educational requirements [66]. In summary, we can say that quantitative research analyzed a wide range of variables using large sample sizes. At the same time, the qualitative studies focused on interpersonal relationships and relationships with money, allowing us to access the symbolic, and to some extent unforeseen, meanings of the BFP.

One point frequently mentioned in qualitative articles is the beneficiaries' capacity to acquire appliances and clothing (e.g., [40]). These goods can directly or indirectly play a fundamental role in improving people's quality of life, including reducing social inequalities by facilitating daily life and increasing the potential for future income, also driven by the education conditionalities (e.g., [42]). In this sense, it can be said that the higher the transfer amount, the greater the likelihood of achieving the program's goals. Beyond the objectives of this review, we identified that qualitative studies indicate that a portion of health professionals has little or no knowledge of the BFP (e.g., [42]). This could impair health services that provide beneficiaries with solutions appropriate to their income context and that support the correct fulfillment of the BFP's conditionalities. In this sense, increased training and/or publicity by the government about BFP, directed at health professionals, could help minimize this lack of understanding.

This scoping review is the second to provide an overview of the impacts of BFP across a wide range of outcomes. The first, conducted by Neves et al. [7], included 57 articles published between 2003 and 2020, and presented limitations, which we endeavored to address. The previous review excluded certain outcomes, was less rigorous in terms of methodology by including studies without a comparison group and did not include qualitative studies. Therefore, we assume that the present review can represent the overall impact of the BFP more accurately. Recent systematic reviews have examined the impacts of the BFP on specific outcomes discussed in this scoping review. For example, Santos et al. [66] identified significant positive impacts on dropout rates and school attendance for girls, but null effect on school performance; and Souza e Heller [77] found reduction in the incidence of illness and death in children under five years of age from diarrhea and malnutrition.

At the same time, different CCTs worldwide have positively impacted the same outcomes addressed in this scoping review. For instance, Moncayo et al. [78] demonstrated that as the coverage provided by Ecuador's CCT Bono de Desarrollo Humano increased, the mortality rate of children under 5 years old due to malnutrition decreased, as well as the overall child hospitalization rate. A report by the Food and Agriculture Organization of the United Nations (FAO) and UNICEF [79] showed that both Zambia's Multiple Categorical Targeting Grant and Kenya's Cash Transfers for Orphans and Vulnerable Children increased household consumption and reduced poverty rates in their respective countries. In Peru, the CCT Juntos increased the chances of school enrollment and finishing primary school among children, according to Gaentzsch et al. [80]. Overall, the broad positive impacts of CCTs worldwide demonstrate the importance of this type of public policy, especially in low- and middle-income countries, helping to improve the life chances of people in social vulnerability [62, 81]. The strength of the present scoping review, compared to broader studies available in the literature, lies in its focus on a specific CCT program (BFP). This approach allows for more detailed and nuanced insights that might be overlooked in broader research. Additionally, this review exclusively includes quantitative studies that evaluated the program's impact using robust statistical analysis, resulting in more accurate and relevant findings.

The main limitation of our study is not covering studies published after March of 2021. As justified in the method, this interruption was made because there was a change in the program's rules in April. In addition, we did not review the risk of bias of the studies, which was not done due to the exploratory nature of the scoping review, and we did not do quality assessment of the studies, which can limit validity of findings. We are aware of a potential for negative publication bias. We did not include grey literature or contact experts, which limits the potential outcomes and coverage of certain areas. However, this was a methodological choice made to ensure adherence to scientific standards. By nature of scoping review, the question was broad and less defined.

Considering our findings and the limitations of our review, new quasi-experimental design studies with rigorous impact analyses on topics not fully covered (gender equality, employability, violence, and teenage pregnancy) are recommended. After addressing the lack of studies in these areas, systematic reviews for critical analysis of findings would be relevant, which is also advised for areas with existing evidence, such as education, poverty, and health. Also, given the increase in the BFP transfer amount in 2023, we suggest that new longitudinal studies be conducted to compare outcomes in beneficiaries' lives before and after the increase in transfer values. The absence of a quality assessment is due to the nature of a scoping review, which, unlike systematic reviews, does not require a quality assessment given its exploratory purpose [82]. Therefore, we recommend that future systematic reviews include these assessments to enable critical analysis and better understanding of current studies. Moreover, given the program's profile, although the government seems to be addressing this issue, there are still no nationally implemented policies for systematic evaluation. These policies would enable the assessment of the program's cost-effectiveness and the identification of the areas in which it is effective.

## Conclusion

The favorable and widespread impacts of BFP in Brazil found by the studies included in this scoping review suggest that the BFP is a public policy that significantly benefits families facing social vulnerability. The timing of this study is particularly relevant considering that between 2014 and 2018 the number of extremely poor people increased by 71.8% in Brazil due to the economic recession, while there was no readjustment of the BFP value according to inflation between 2015 and 2017 [3]. In this context, the literature on the BFP impact suggests that the BFP may be even more essential for beneficiaries. As extreme poverty and poverty began to rise again in Brazil in 2021 [3], the BFP has become even more crucial, standing out as an effective method to combat social vulnerability [5].

## Supplementary Information

Supplementary Material 1.

## Data Availability

The datasets generated during the current study are available in the OSF repository, https://doi.org/10.17605/OSF.IO/WG2P7. The datasets analyzed during the current study (spreadsheet with 1,546 studies identified via databases) are available from the corresponding author on reasonable request.

## Abbreviations

JM: Júlia Magalhães
CZ: Carolina Ziebold
AM: Alicia Matijasevich
CSP: Cristiane Silvestre Paula
SE-L: Sara Evans-Lacko
FAPESP: São Paulo Research Foundation
CAPES: *Coordenação de Aperfeiçoamento de Pessoal de Nível Superior*
CAPES/PrInt: *Coordenação de Aperfeiçoamento de Pessoal de Nível Superior/Programa Institucional de Internacionalização*
CTP: Cash Transfer Programs
BFP: *Bolsa Família* Program
OSF: Open Science Framework
ERIC: Education Resources Information Center
ECOS: National Health Economics Information Portal
SciELO: Scientific Electronic Library Online
PICO: Population, intervention, comparison, and outcomes
NP: Not presented
HDI: Human Development Index
IPEA: Institute for Applied Economic Research
PNAD: National Household Sample Survey
IBGE: Brazilian Institute of Geography and Statistics
LAZ: Height-and wright -age z-score
WAZ: Weight-for-age z-score
CBT: Cognitive behavioral therapy
CNPq: National Council for Scientific and Technological Development

## Footnotes

1 Dollar value at BRL 5.55, updated for July 22, 2024.

2 Federal government website page explaining the task force created: https://www.gov.br/mds/pt-br/servicos/sagi/avaliacao-de-impactos-economicos-dos-programas-sociais-programa-bolsa-familia

3 Dollar value at BRL 5.55, updated for July 22, 2024.

4 Handbook developed by the Federal Government on the BFP: chrome-extension://efaidnbmnnnibpcajpcglclefindmkaj/https://www.mds.gov.br/webarquivos/MDS/2_Acoes_e_Programas/Bolsa_Familia/Cartilha/Cartilha_Bolsa_Familia.pdf
