# Supplementary material for: Health, economic and social impacts of the Brazilian cash transfer program on the lives of its beneficiaries: a scoping review
Source: BMC Public Health. 2024 Oct 14;24:2818. doi: 10.1186/s12889-024-20046-2 (PMC11476833; doi:10.1186/s12889-024-20046-2)
Supplement: Supplementary file 1 — Supplementary Material 1. [file 12889_2024_20046_MOESM1_ESM.docx]

**Supplementary Material**

**Supplementary table 1.** Main characteristics of the included articles (n=94).

| **Author and year** | **Topic addressed** | **Study aims** | **Design** | **Participants** | **Sample size** | **Geographic location** | **Primary outcome** | **Main results** |
| --- | --- | --- | --- | --- | --- | --- | --- | --- |
| Alejo, Bérgolo & Carbajal (2014) | Poverty | To analyze the effect of household income sources, and in particular the contribution of public transfers, on changes in income inequality in the countries of the Southern Cone of Latin America in the 2000s. | Non-parametric decomposition methodology | Information not clear | Information not clear | Nacional: Brazil, Argentina, Chile and Uruguay | Inequality in terms of household income (per capita). | Non-labor income had a significant contribution to changes in inequality. The effect on inequality were strongly associated with the implementation or expansion of non-contributory transfer programs in the past decade. |
| Almeida, Mesquita & Silva (2016) | Poverty | To analyze the effect of the Bolsa Familia Program (PBF) on the level of diversification of food consumption among beneficiary families residing in urban areas of Brazil, also considering nutritional properties of the consumption basket. | Cross-sectional | Information not clear | 33,255 families | Nacional: urban areas of Brazil | Level of healthy diversification of food consumption. Quantity; distribution; and nutritional weight. | The BFP presented a positive result on the level of consumption for a basket with more variety of food items, but it fails to contribute to changes in terms of a basket diversified by more healthy items. |
| Alves & Escorel (2013) | Poverty | To understand the impact of Bolsa Família (PBF), a federal cash transfer program, and to analyze its effects on social inclusion and exclusion processes experienced by low-income families in Brazil, with a focus on the program’s potential to help overcome health inequity. | Qualitative | Adults > 20 years | 31 households | Rio de Janeiro: Silva Jardim | Perceived social and economic inclusion. | BFP reduced social and economic vulnerability by increasing consumption. BFP improved self-valuation of the beneficiary as a consumer or as someone who was able to pay his bills on time. |
| Alves, Machado & Barreto (2019) | Violence | To assess the effect of PBF coverage on suicide rates in Brazil. | Ecological longitudinal study | Information not clear | 5,507 Brazilian municipalities | Nacional: Brazilian municipalities | Suicide, defined as the cause of death recorded as “intentional self-harm”. | An increase in the duration of high PBF coverage of 70% or more was associated with a fall in suicide rates. |
| Amaral & Monteiro (2013) | Education | It analyzes the impact of the educational conditions of Brazil's Bolsa Família Program on the dropout rates of children benefiting from the program. | Ecological study | People from 7 to 14 years old | 9,232 children | Nacional: Norte, Centro-Oeste, Nordeste, Sul and Sudeste | The chance that children would drop out of school. | The receipt of the Bolsa Família Program benefit proved to be responsible for a reduction in the chances of dropping out of school. |
| Andrade et al. (2012) | Health | It investigates the impact of the Bolsa Família Program on the immunization of children from 0 to 6 years of age in Brazil and its regions. | Case-control | Children from 0 to 6 years old | 7,550 children | Nacional: all regions of the country | Immunization records of children. | The comparisons of the Bolsa Família Program beneficiaries and non-beneficiaries showed that there are no statistical differences in immunization coverage. |
| Araujo, Alves & Besarria (2013) | Poverty | To analyze the effects of social spending on poverty indicators and indexes of income inequality in the period 2004-2009. | Econometric panel data evaluation | All Brazilian states | 27 federal units | Nacional: all regions of the country | Number of extremely poor people, number of poor people, Theil index, Gini index, GDP increase rate. | BFP decreased poverty indicators related to the Brazilian states. However, no evidence was found that BFP have produced statistically significant effects on the rates of income inequality. |
| Barrientos, Debowicz & Woolard (2016) | Employability | The paper examines heterogeneity in program outcomes from *Bolsa Família*. | First part: scope review; Second part: quantile regression model | People from 18 to 60; people in school age from 6 to 15 years old | 273 municipalities | Nacional: all regions of the country | Adult labour participation and regular school attendance. | BFP is not associated with changes of statistical significance in labour force participation among adults. There were positive effects of BFP on the school attendance of girls aged 6–15, but significance varies across quantiles. |
| Bohn et al. (2014) | Poverty | The paper examines whether the state, through conditional cash transfer programs (CCT), can reduce the poverty and extremely poverty in societies marred by high levels of income concentration. | Mixed method approach; retrospective study | People 16 years or older | Quantitative survey: 4,000 beneficiaries; Qualitative survey: 38 beneficiaries | Nacional: all regions of the country | The attainment of food security; access to the educational system and the acquisition of professional qualifications; entry into the job market. | Those who before their participation on PBF were at the margins have now been able to access healthcare services on a more regular basis. BFP seemed to decrease the food insecurity in very five years. |
| Camargo & Pazello (2014) | Education | To evaluate the effect of an increase in the percentage of students treated by the BFP in each school on the average performance of that same school. | Quasi-experimental | 3rd to 4th grading students | Information not provided | National | Average rates of approval and dropout of schools in 2009 as well as on the average proficiency of schools in *Prova Brasil.* | An increase in the proportion of students treated in a school, on average, reduces school dropout rates. It was not possible to say that a higher proportion of students benefited leads to a reduction in approval. |
| Campoli et al. (2020) | Poverty | To measure the efficiency of the Brazilian Federative Units (FU) in converting government expenditures of the Bolsa Familia Program to advance toward Millennium Development Goals | Econometric analysis-Panel data | 27 federal units | 27 federal units | National | Income of poor people; Gini index; School attendance; Child mortality rate; Maternal mortality. Life expectancy. | Because of BFP, the income of poor people showed increase 2.62% in reason of BFP. Gini index decreased 0.35%; School attendance in elementary school raised 0.17%; Infant mortality decreased 1.82%; Maternal mortality increased in consequence of BFP; and the life expectancy enlarged 0.45%. |
| Carter, et al. (2019) | Health | To estimate the impact of a conditional cash transfer programme on TB treatment success rates. | Propensity score matching | Information not clear | 2,167 individuals | Information not clear | The ‘treatment success’ outcome includes those who completed treatment with or without bacteriological confirmation | This study further confirms a positive relationship between the provision of conditional cash transfers and TB treatment success rate. |
| Carvalho et al. (2014) | Health | To present the perception of professionals from Family Health teams in municipalities in northeastern Brazil about the changes in the lives of families participating in the Bolsa Família program, their relationship with health services and the impact on the work dynamics of professionals, based on the monitoring of the health conditionalities of the Bolsa Família program. | Qualitative, exploratory | Majority between 21 and 30 years | 53 professionals | Tibau do Sul-RN, e Barra de São Miguel-AL | Changes caused in the lives of the families participating in the BFP; changes in the relationship of the families participating in the PBF with the health services; and impact on the work dynamics of the professionals. | The program led to poverty reduction, increased school attendance of children and positive changes in the relationship between participating families and health services. |
| Carvalho et al. (2015) | Poverty | This paper studies the impacts of Bolsa Família on dairy products demand. | Quasi-experimental | 20–65 | 32,371 households | Nacional | Expenditure on the good and its quantity purchased. | This finding indicates that Bolsa Família has not being used to purchase dairy products other than the goods that compound the group other (milk powder, butter, cream etc.). |
| Cavalcanti et al. (2013) | Poverty | To verify the impact of the Bolsa Família Program (PBF) for families in northeastern Brazil, on its main goals: immediate poverty/income inequality relief and school attendance. | Propensity score matching | <17 | 12,862 children in 2004; 12,205 children in 2006 | Rural and urban sector of the Northeast | Number of children and young people up to 17 years attending school and effect of the program on total family income. | There was a positive impact of the programme on the income of the beneficiaries, especially the extremely poor - as well as the Foster-Greer Thobecker (FGT), which exposed a slight improvement in the proportion of poor people and inequality among the poor, however the magnitude of these effects was low. |
| Cecchin & Parente (2019) | Gender equality | To smooth out the effects of the Bolsa Família Program on gender relations and women's empowerment at *Acampamento Ilha Verde*, located in Babaçulândia, TO. | Qualitative with inductive analysis | 24–48 | 7 women | Babaçulândia, TO | Women's empowerment in the personal, interpersonal, and collective dimensions. | In the collective dimension, there was a lack of participation in the decision-making arenas for the beneficiaries of the Bolsa Família Program, such as the Municipal Council of Social Assistance or the SUAS Users' Forum, mentioned throughout the article. |
| Cechin et al. (2015) | Teenage pregnancy | Investigates a possible incentive from the Bolsa Família Program to increase the fertility of its beneficiaries due to its rules, according to which the amount of funds transferred depends on the number of family children. | Propensity score matching | 16–49 | 3,013,896 women | National | Fertility of the beneficiaries. | The Midwest region obtained the highest value of the effect, presenting an ATT of 4.65 percentage points, that is, a beneficiary of Bolsa Família is 4.65 percentage points more likely to generate the second child compared to her control group match. |
| Chioda et al. (2016) | Violence | Estimates the contemporaneous effect of the Bolsa Família program on crime. | Quasi-experimental | 16–17 | 581 high schools | São Paulo, SP | Crime: place of occurrence (latitude and longitude), type of crime, estimated time of occurrence, and, sometimes, characteristics of the suspected offender (such as age and gender). | There is a robust and significant negative impact of Bolsa Família on crime. |
| Chitolina et al. (2016) | Employability | Evaluates the impact of the expansion of the Bolsa Família program to families with youths aged 16 to 17 years on the time allocation of youths and on the labor supply of their parents. The effects of the benefit were investigated with regard to the school enrollment of beneficiary youths and also to their time allocation decisions in terms of working and studying activities. | Quasi-experimental | 16–17 | Information not clear | Nacional | Allocation of the time of young people and on the offer of work of their parents. | The program hardly impacted the parents' labor supply decisions. These results confirm what has been previously found in the literature on the absence of a "laziness effect" of cct programs. |
| Coelho & Melo (2017) | Poverty | This article analyzes the impact of the Bolsa Família Program (PBF) on the diet quality of households in the state of Pernambuco using data from the Family Budget Survey (POF-IBGE) between 2008-2009. | Quasi-experimental | Information not provided | 518 family units | State of Pernambuco | Diet quality index. | There is efficacy of PBF in increasing the quality of the diet of families, mainly regarding the reduction of fat and sodium, and increase of variety. |
| Correa Junior et al. (2019) | Employability | To investigate the impacts of PBF on the formal labor market of 5,570 Brazilian municipalities, from 2004 to 2013. | Quasi-experimental | Not applicable | 5,570 municipalities | National | Number of formally employed salaried personnel and the total of salaries and other compensations. | There was a positive association between the BFP benefits in the municipality and the salaried employed personnel, the salaries and other incomes. |
| Costa et al. (2018) | Employability | Evaluates the effects of the Bolsa Família Program (PBF) on the labor market of poor families living in rural areas of Brazil. | Propensity score matching | Not specified | 430,227 home units | National | Labor market was assessed using worked hours of the head of household and income from labor per capita. | There were neutral effects on quants where the hours worked are equivalent to a maximum of 3 hours for all regions, as well as in the quantities where the hours worked are 40 hours, with the exception of the Midwest region; Negative effects, especially where hours worked are longer than 40 hours, perhaps explained by the increase in the worker's bargaining power to forgo excessive working hours; Positive effects between 0.1 and 0.25 in the North. |
| Costa-Fernandez & Munoz (2019) | Gender equality | Present a brief theoretical review and some partial results of a master's thesis held at ppg in psychology at the Federal University of Pernambuco (UFPE). Qualitative design, the study aims to analyze the discursive practices that constitute the modes of subjectivation of women beneficiaries of the Bolsa Família Program (PBF) in the rural context of the Zona da Mata Sul Pernambucana. | Qualitative, critical analysis of the discourse | >18 | 6 women | Zona da Mata Sul Pernambucana | Three major themes ("Family Reality", "Rural Context" and "Bolsa Família Program"). | BFP is considered a fixed and safe income, predictability that gives the possibility of planning, a "near salary" for the woman. It is also perceived in the narratives a high dependence on the benefit and the permanent fear of losing it. For the interviewees, the amount received should be used in favor of the children. To receive the benefit brings important changes for women, such as greater financial autonomy and improvement of self-esteem. |
| Da Mota et al. 2014. | Gender equality | To analyze the relationship between the organization of family work in extractivism and the participation of extractivists in public policy programs in the state of Sergipe. | Qualitative with the use of non-directive interviews | Information not clear | 50 women | Coastal towns of Sergipe | Meanings attributed by the subjects to the processes they experience about extractivism in fishing. | BFP reinforces the traditional roles of men and women. The program reinforces the greater presence of women in the domestic sphere and, thus, reduces their vulnerability to offer almost free labor in the labor market. |
| De Andrade et al. (2018) | Health | To assess the effect of the Brazilian conditional cash transfer programme on the reduction of new case detection rates (NCDR) of leprosy in Brazilians under 15 years old. | Mixed ecological study, fixed effects negative binomial models for panel data | <15 | 1,120 municipalities | North, Northeast and Central-West regions of the country | New case detection rate of leprosy. Annual new case detection rate of leprosy in individuals younger than 15 years old, calculated as the number of new leprosy cases detected in people under this age group, per 100,000 inhabitants. | BFP was associated with a reduction of the New Case Detection Rate of leprosy among Brazilians aged less than 15 years old, living in municipalities with a high risk of leprosy transmission. |
| De Brauw et al. (2014) | Gender equality | We interpret the municipality-level variation in procedures as suggesting that, once a large set of household characteristics and municipality characteristics are accounted for, the probability that a particular household is a recipient of Bolsa Família is uncorrelated with the outcomes we consider. | Quasi-experimental | Information not provided | 6,838 households | National | Questions in our dataset that directly address the process of decision making. Women were asked questions about who in the household generally makes decisions about a range of issues. | Bolsa Família had meaningful impacts on women’s control over decision making, but with considerable heterogeneity. |
| De Brauw et al. (2015) | Education | To assess the impact of Brazil’s Bolsa Família conditional cash transfer program on schooling outcomes of children aged 6–17 years. | Quasi-experimental | 6–17 | 6,056 children and adolescents | National | Questions about whether the child currently participates in school; has progressed from the previous grade level; participating in school the previous year, repeated the previous grade level and dropped out. | Bolsa Família increases girls’ school participation by 8.2 percentage points with comparable effects for younger and older girls. There is no impact on boys’ participation. Bolsa Família has a large effect on grade progression for all girls living in rural areas with the effect larger for girls aged 15–17 (22.5 percentage points) than girls aged 6–14 (14.6 percentage points). |
| De Senna & Souza (2016) | Poverty | To analyze the relationship between the main macroeconomic variables and the Federal government spending on social welfare policy in the period from January 2004 to August 2014. | Quasi-experimental | Not applicable | 126 observations | National | Macroeconomic variables, such as: Activity Rate, Inflation Rate by the Broad Consumer Price Index, Selic rate, the Personal income expectancy rate or Income Rate, the ICEA consumption index, Current Economic Conditions Index, Industrial Production Index, the Unemployment Rate, Real Minimum Salary and Gross Domestic Product. | We found in this study that the macroeconomic variables are affected by innovation in social assistance programs and their effects are transmitted as time passes. |
| De Souza et al. (2018) | Health | To evaluate the impact of the Family Health Strategy (FHS) and the Bolsa Família Programme on TB-related mortality in Brazil | Ecological study | Not applicable | 1,614 municipalities | National | TB mortality rate. | An increase in the coverage of the Brazilian cash transfer programme was significantly associated with a reduction in the TB mortality rate (RR 0.87, 95%CI 0.81–0.96). |
| Duarte et al., (2009) | Poverty | Evaluates the impact of the transfer of income from the Bolsa Família Program on food expenditures of rural families. | Quasi-experimental study | 18–93 | 822 families | Paraíba (Cariri), Ceará (Sertão Central), Rio Grande do Norte (Apodi) and Sergipe (Sertão) | Expenditure on food consumption of each individual was aggregated to the family unit. | BFP has a positive impact on the food consumption of selected families. |
| Dyngeland et al. (2020) | Poverty | To assess how Brazil’s flagship Zero Hunger (ZH) social protection have affected outcomes related to sustainable development goals (SDGS): food production, multidimensional poverty, child malnutrition, infant mortality, and changes in natural vegetation cover. | Quasi-experimental design | Not applicable | 3,786–4,976 rural municipalities across Brazil (74–97% of all) rural municipalities) | National | Impact on changes in multidimensional poverty, food production and child malnutrition. | BFP increased protein production. We find four states with BF-linked reductions in kilocalorie production and two states with BF linked kilocalorie increases. BF had limited capacity to alleviate multidimensional poverty and, in some regions, is associated with increased poverty. We do find that BF investment is associated with improvements in the educational dimension. BF investment is associated with increased SIAB derived infant mortality. |
| Faria et al. (2020) | Poverty | To reflect to which extent access to this program that was adopted to combat poverty, may represent a structuring factor of possibilities for livelihood diversification strategies | Qualitative | Unspecified | 16 Families and representatives of 3 services related with BFP | Rural area of ​​the municipality of Luminárias, state of Minas Gerais | The perception of beneficiaries in relation to the program and the main changes observed from access to the program. | The monetary resources from the BFP represented a fundamental contribution to meeting some basic needs of the analyzed families with relative freedom of consumption. Also contributed to the expansion of inclusion/integration in spaces of sociability. Contributed to the minimization of vulnerabilities and deprivations. Courses for beneficiaries were offered at the city's headquarters. |
| Garmany (2016) | Poverty | Examines the geographic effects of conditional cash transfer programs | Qualitative | Information not clear | 45 people | Sobral, CE, and Parnaíba, PI | Questions to residents focused on the daily challenges they confronted, municipal infrastructure needs and future concerns, their engagements with citizenship and interactions with the State more generally. 3. ‘expert’ opinions on the subject matter. | BFP has introduced new economic practices, migration patterns, trends in urban growth, infrastructural projects, political strategies, and so on. Local residents now have better access to schools, health clinics, formal markets, and transportation networks, and due to PBF their engagements with the State have expanded in many respects. |
| Glewwe & Kassouf (2012) | Education | To evaluate the impact ofbolsa Escola/Familiaon enrollment, dropping out and grade promotionat the primary and lower secondary levels. Both school andmunicipio(county) level estimates are presented. | Quasi-experimental design | Schools with grades 1–8 | 1-4 grades 698,229 schools 5-8 grades 182,007 schools | Brazil | Changes in enrollment, dropping out and grade promotion across schools that adopted BFP. | The program increased enrollment by about 5.5% in grades 1–4 and 6.5% in grades 5–8, decreased dropout rates by about0.5 percentage points in grades 1–4 and 0.4% in grades 5–8, and raised grade promotion rates by about 0.9 percentage points in grades 1–4 and 0.3 percentage points in grades 5–8. |
| Guanais (2015) | Health | To examine the combined effects of access to primary care through the Family Health Program (FHP) and conditional cash transfers from the Bolsa Familia Program (BFP) on postneonatal infant mortality (PNIM) in Brazil. | Longitudinal ecological analysis | Preschoolers | 4,583 Brazilian municipalities | Brazil | Postneonatal infant mortality (PNIM) rate. | The association of higher FHP coverage with lower PNIM became stronger as BFP coverage increased. |
| Hecktheuer, Souza & Hecktheuer (2018) | Poverty | To reflect whether the Bolsa Família Program is an efficient public policy in the expansion of the substantial freedoms of individuals. | Qualitative | 18–60 | 22 women heads of family | Distrito de São Carlos, located in the municipality of Porto Velho, Rondônia | The importance of the BFP for children's health and schooling; we also sought to understand the importance of the PBF for the family and where the resources are used. | In all interviews, the beneficiary was a woman, who reported the great importance that the program has for feeding their families, as well as for the purchase of items needed for food, as well as for bearing the costs of acquiring items from outside the community, such as school materials and clothing, since most of these families do not have a fixed income. |
| Hone et al. (2019) | Health | To assess the association between economic recession and adult mortality in Brazil and to ascertain whether health and social welfare programmes in the country had a protective effect against the negative impact of this recession. | Panel regression methods | ≥15 | 5,565 Brazilian municipalities | National | Mortality rates were calculated for all causes and for 17 selected causes of death based on WHO classifications and calculated all-cause mortality rates. | Increases in overall mortality in municipalities in the lowest terciles (0·78 increase per 100 000 population [95% CI 0·38–1·18]) and middle terciles (0·61 [0·15–1·08]) of Bolsa Familia expenditure per poor person, and in municipalities with the lowest public health expenditure per capita (0·63 increase per 100 000 population [0·24–1·02]. |
| Hone et al. (2020) | Health | To assess the association of PHC usage and mortality of low-income adults and whether there are differences across socioeconomic groups and causes of deaths | Quasi-experimental | Adults (aged 15–84 years) | 1,241,351 adults | Rio de Janeiro city, Brazil | Time-to-death from all-causes and selected causes. | There were greater reductions in the risk of death for FHS users who were in receipt of conditional cash transfers (Bolsa Família) (HR 0.51, 95% CI 0.49–0.54, p < 0.001) compared with nonrecipients (HR 0.63, 95% CI 0.60–0.67, p < 0.001). |
| Labrecque et al. (2018) | Health | To estimate the effect of the Brazilian conditional cash transfer program, Bolsa Família (BF), on child nutritional status as measured by length-for-age z-score (LAZ) and weight-for-age z-score (WAZ) at 24 months. | Quasi-experimental study | Newborns within 24h of birth | 1,703 families | Pelotas, RS | Height-for-age (HAZ) or length-for-age (LAZ) at 12 and 24 months. | BFP was associated with a reduction in LAZ and WAZ in 24-month-old children. |
| Lima et al. (2017) | Poverty | To analyze the impacts of PBF on the socioeconomic context of beneficiary families living in the rural community of Sítio Carnaubal in the municipality of Água Nova, a small city of Rio Grande do Norte (RN), a state located in the northeast region of Brazil. | Information not clear | Information not provided | 25 women | Água Nova, RN | Socioeconomic context. | When the BFP was territorialized, the territory itself was transfigured as families experienced transformations in their daily realities: less beset by poverty, new possibilities were created or, being less optimistic, impossibilities were reduced. |
| Litwin et al. (2019) | Gender equality | To explore how a conditional cash transfer (CCT) may influence intimate partner violence (IPV). | Qualitative and quasi-experimental | 15–49 | Qualitative: 13 people | Information not clear | The prevalence of violence in their communities, female empowerment, relationship and household roles. Quantitative: female homicides. | Seven out of 13 respondents, including four community members and three service providers, reported that the CCT increases intimate partner violence. Quantitative results suggest that at a national level as well as for subpopulations, Bolsa Familia has not had an impact on female homicides. |
| Machado et al. (2018) | Violence | To investigate the potential effect of the BFP on homicide rates and on hospitalizations from violence in Brazil. | Mixed ecological design | All ages | 5,507 Brazilian municipalities between 2004 and 2012 | Secondary data, all regions of the country | Homicide and hospitalization rates. Homicide rate. | Homicide rates and hospitalization from violence decreased as BFP coverage increased in the target population. |
| Magalhães et al. (2011) | Gender equality | To analyze the perception of women in situations of social vulnerability, about their dreams and the ways of coping with the adversities present in their daily lives, seeking to perceive how the situation of poverty, according to The Conceptions of Amartya Sen, interfered in their attitude towards life. | Qualitative, thematic analysis technique | 31,6% were aged between 20 and 39 | 68 women | Paula Cândido, MG | We sought to understand what the women interviewed valued in their lives; their future prospects; their main strategies to fight for what they wanted, as well as to face everyday difficulties. | It is not enough to promote the sustainable emancipation of fmailias and the integral fulfillment of their needs. |
| Mariano & Carloto (2013) | Gender equality | Discuss theresults of a survey conducted with women holders of the Bolsa Família Program (PBF) in Uberlândia-MG and Londrina-PR, in order to analyze possible variations related to the condition of inequality, taking into account the color/race of the respondents. | Qualitative | 72 declared themselves brown and black, i.e. 70.6% | 102 women | Uberlândia-MG and Londrina-PR | Variations related to race/ethnicity related to topics dealing with schooling, income, work and female family management. | PBF exerts more influence on the daily lives of black women compared to white women. However, this influence does not generate the impact of equalizing the situation between these two groups of women. |
| Marins et al. (2018) | Gender equality | To analyze, under the gender bias, how is developed the construction of moral and symbolic boundaries resulting from Bolsa Família. To clarify the properties of these borders and the ways of negotiating their content. To beter understand the construction of the status of beneficiaries (mothers). | Qualitative | Children, adolescents, adults | Not reported | Itaboraí, Rio de Janeiro State, Brazil | Views about 'who deserve the benefit' and the perception of 'being controlled by the state. | The relations established between beneficiaries and non-beneficiaries (institutional stakeholders and neighboors) are based on a moral matrix (good mothers, extremely pooor people, older people, higher number of children, deserve the benefit), with the presence of gossip (seen as freeloader), moral judgments and specific social controls that are developed around the figure of the beneficiary. |
| Marins (2014) | Poverty | To analyze the sociopolitical representations and daily practices of actors involved in some way in the performance of the State, in particular, through a policy of income transfer: the Bolsa Família Program | Qualitative | Information not clear | Not reported | Itaboraí, Rio de Janeiro State, Brazil | Sociopolitical representations and the daily practices of actors involved in some way in the performance of the State; As two antagonistic repertoires (rational/legal and emotional) are applied contextually by registrants, Social Workers and Health Agents. | The target population of such program (the poor) also react by using moral tools that justify they are deserving of the benefit and others are not, thus reinforcing moral repertoires (merit and honor) rather than civic ones (rights). |
| Martins & Monteiro (2016) | Poverty | To assess the impact of the Bolsa Família Program on food purchases of low-income households in Brazil. | Quasi-experimental | All ages | 11,282 households | All regions of the country | The purchases of food items for household consumption by the families for seven consecutive days, recorded daily in a notebook, by household members or IBGE interviewer if necessary. | Compared to non-beneficiaries, the beneficiaries’ households had 6 % higher food expenditure (p = 0.015) and 9.4 % higher total energy availability (p = 0.010). It was found a 7.3 % higher expenditure on in natura or minimally processed foods and 10.4 % higher expenditure on culinary ingredients among the Bolsa Família Program families. |
| Mattar, Monteiro & Azize et al. (2021) | Poverty | The article discusses how the Bolsa Família Program (PBF) is mobilized and gains different meanings in the practices and discourses of beneficiaries in a favela in Rio de Janeiro | Qualitative | Information not clear | Not reported | Favela do Rio de Janeiro | Broader issues related to this public policy, such as feedback between precariousness and surveillance, constitutive dimensions of slum life. | Expectations about female beneficiaries reaffirm gender conventions and make evident the moralization of the female place and maternal condition. In addition to state violence and organized crime agents, diffuse controls and surveillance, the other dimension of residents' sociability concerns vulnerability and precariousness. |
| Mello (2020) | Employability | To discuss the possible impacts of the Bolsa Família Program on the labor insertion of its beneficiaries in the city of São Luís | Qualitative | 14 years or older - not detailed (the majority were female) | 28 | São Luís municipality (Maranhão State) | Insertion in the labor market. | All Beneficiaries stated that no change occurred regarding insertion in the labor market after inclusion in the program. The amount received is small and insufficient to support a family and, therefore, does not dispense with the need to look for a job. |
| Melo & Duarte (2010) | Education | To evaluate the impact of the Bolsa Família conditioned public cash transfer program on the school presence among the children and adolescents from five to 14 years, in the Brazilian states of Pernambuco, Ceará, Sergipe and Paraíba | Cross-sectional | 5 to 14 | 745 children | PE, CE, SE, PB | School attendance of students. | Comparing the school attendance of the treatment group with the control of the field sample, it is noted that the school attendance is higher for the group of children who belong to the families benefited by Bolsa Família (98.1%). |
| Moreira et al. (2016) | Gender equality | Analyze how the Bolsa Família Program is able to influence domestic violence. | Quasi-experimental study | Information not clear | 477 women | Information not clear | Characteristics of victimization and access to justice in Brazil and risk of domestic violence against women. | The PBF has the effect of increasing violence against women. Moreover, those domiciled in rural areas, with low schooling and with relatively lower incomes than spouses are those that deserve more attention, since this group has a higher risk of suffering violence. |
| Moreira et al. (2012) | Gender equality | To analyze the empowerment of the women who are beneficiary from the “Programa Bolsa Família” under the social agents´ perception in the Social Assistance Reference Centers (Cras). | Qualitative designs. Multiple-case study | Adults | 11 CRAS managers | Minas Gerais State | Women empowerment. | Cras are important for either execution of the “Programa Bolsa Família” and the empowerment process, since the participation in this location have contributed to the awareness of the rights for the social insertion and the improvement of the women’s welfare. On the agents’ perception, it was possible to observe the improvement in life conditions, family relationships, awareness and self-esteem. |
| Morton (2019) | Gender equality | To investigate the everyday uses of money that women receive from two gender-targeted social programs in rural Brazil, Bolsa Família and Maternity Wage | Ethnographic research | Adults | 96 households. 51 bfp beneficiaries, 12 had received maternity wage. 9 households were selected to conduct focus family interviews | Maracujá and Río Branco villages: Rural area of Vitória da Conqusita, Bahia, Brazil. | Assets acquisitions using CT money. Modeled on the PNAD, the census asked about each household’s earnings and assets for 2011. | Women typically spend monthly cash assistance on durable items, like clothing and furniture, that correspond to local stereotypes about feminine property. Monthly money reinforces gendered stereotypes about assets, while lump-sum money challenges those stereotypes. Lump sum payments may be an effective way to combat gender inequality in asset ownership. I identify two key qualities that underlie this change: a payment’s large size and its unpredictability. |
| Nanes & Quadros (2018) | Gender equality | We analyzed repercussions of this ownership on the productive trajectories of 12 women living in Coque. We describe important aspects of productive trajectories, marked by formal, vexatious and degrading work. | Anthropological research | 20–30 | 12 women | Recife, PE | (I) reproductive rights and motherhood; (ii) productive insertion/qualification, agency strategies and autonomy/empowerment processes and (iii) life projects (related to expansion of choices for agency and female empowerment). | The insertion in the PBF did not accommodate women in the search for work, demystifying the discourse of the laziness effect. |
| Nascimento et al. (2017) | Poverty | To analyze the role that Programa Bolsa Família plays in the food security of the families in the Marajó Territory, located in the State of Pará, Brazil | Qualitative study | Adults | 50 adults | Five municipalities of Marajó Territory, located in the State of Pará, Brazil: Cachoeira do Arari, Salvaterra, Curralinho, Melgaço e Portel | Food insecurity. | A positive impact was observed in access to food and standard of living, providing well-being, beter quality of life and development of credit practices, necessary to combat food insecurity in the region. |
| Neri & Osorio (2019) | Education | Analyze the impact of the Bolsa Família Program (PBF) on time at school, its components (enrollment, presence and journey) and on the motivations related from the special supplements of the National Household Sample Survey (PNAD) of 2004 and 2006. | Quasi-experimental study | 6–15 | Information not provided | National | Indicator that assign the length of the workload to which each student is subjected daily in school, incorporating the classroom attendance of enrolled students, in addition to the zero hour of study of potential students who are out of school. | Marginally eligible young people of age to the PBF had 62%, 8% and 6% higher chances of attending school, attending the minimum attendance and studying more than 4 hours a day, respectively, than the almost eligible ones. Finally, tests on the alleged motivations for transgressing school conditionalities do not rule out a current income relief effect provided by the PBF. |
| Nery et al. (2017) | Health | To evaluate the impact of the Brazilian cash transfer programme (BFP) on tuberculosis (TB) incidence in Brazil from 2004 to 2012. | Ecological multiple-group and time-trend design | Not applicable | 2,458 municipalities | National | Annual incidence TB rate, which was calculated as the number of new TB cases all forms notified (defined by the codes A15–A19 of the International Classification of Diseases, tenth edition) per 100 000 population. | TB incidence rates were significantly reduced in municipalities with high BFP coverage compared with those with low and intermediate coverage. |
| Nery et al. (2014) | Health | To evaluate the impact of the Brazilian cash transfer (Bolsa Família Program-BFP) and primary health care (Family Health Program-FHP) programs on new case detection rate of leprosy. | Ecological multiple-group and time-trend design, Longitudinal panel data models | <15 | 1,358 municipalities | Nacional | The annual new case detection rate of leprosy (NCDR), was calculated as the number of reported new cases of leprosy (defined by the code A30 in the International Classification of Diseases – 10^th^ revision), per 100,000 people. | BFP was associated with significant reduction in the NCDR of leprosy. |
| Nunes & Mariano (2015) | Employability | To investigate whether social and cash transfer programs affect children's and parents' decisions to participate and allocate hours of work in non-agricultural activities. | Cross-sectional | Adults | Not provided. | Rural areas of northeast | Decisions to participate and allocate working hours in non-agricultural activities. Decision to work (in non-agricultural activities = 1; in agricultural activities = 0). Worked hours (hours / week in non-agricultural activities). | Negative effect of income transfer programs and social programs on the decision of parents and children to participate in non-agricultural activities. |
| Oliosi et al. (2019) | Health | To evaluate the independent effect of the Bolsa Familia Programme (BFP) on tuberculosis treatment outcomes in Brazil. | Prospective cohort study | ≥18 | 1,239 adults | Information not provided | Cure, dropout, death, or development of drug-resistant tuberculosis or treatment failure was recorded after 6 months of therapy. | BFP alone had a direct effect on tuberculosis treatment outcome and could greatly contribute to the goals of the WHO End TB Strategy. |
| Olson, Clark & Reynolds (2018) | Teenage pregnancy | To estimate the impact of this policy on teen fertility with a triple difference analysis on the fertility outcomes of treated cohorts vs. Non-treated cohorts based on income eligibility, age eligibility, and timing of program implementation. | Quasi-experimental, triple difference estimation strategy | 16–19 | Information not clear | National | Whether or not a teenager was ever pregnant. This is a dummy variable with a value of 1 if a woman has any children and 0 if she does not. | This study shows that Bolsa Família’s CCT targeted at teens reduced teen fertility among the urban poor by 10% within 5 years in Brazil, bringing fertility rates for adolescents living in poverty down to nearly the same rates as other adolescents in Brazil. |
| Passos & Waltenberg (2016) | Gender equality | To measure, based on data from PNAD 2006, the effects of being a beneficiary of the PBF on the “individualization” of women, taking proxies the female journey in the labor market and the domestic work journey. | Cross-sectional | Ecological study | Not provided. | National | Domestic work (Weekly hours spent on domestic activities), participation in the labor market (Tells whether the individual is economically active) and Weekly hours spent in paid work (Paid working hours), for men and women. | BFP does not contribute to the “individualization” of poor women, but on the contrary, it reinforces traditional roles that associate women with home care and family responsibilities, since the group of beneficiaries, compared to that of non-beneficiaries in a similar situation , has a greater dedication to unpaid work (domestic care) and less time in paid work, while men in the beneficiary household devote less time to care. |
| Peixoto et al. (2011) | Poverty | Analyze the significance of PBF conditionalities for its beneficiaries. | Qualitative, sampling was intentional, delimited by the saturation method | 20–40 | 24 women | Campina Grande, PB | Meanings of the PBF in the lives of beneficiaries. | The PBF is an important initiative and that the regularity of receiving the benefit has contributed to the beneficiary families being able to program their expenses in favor of better family satisfaction; favors the purchase of consumer goods, incentives to attend health and education services, as well as access to specialized health services and medicines that are not available in the public health network. |
| Peixoto et al. (2011b) | Poverty | The objective of this study is to investigate the meaning of the FGP to its beneficiaries. | Qualitative and descriptive | 20–40 | 24 women | Neighborhood of the Pedregal, Campina Grande, PB | Meanings of the PBF for its beneficiaries. | The relationship that the beneficiaries of this study make between the benefit of the PBF and the improvement of basic needs is the possibility of offering their children consumer goods that they did not have the opportunity to acquire throughout their lives and the guarantee of income in the absence of paid activity. |
| Peña et al. (2015) | Poverty | To analyze the trend and mobility in distribution income before and after the PBF, in order to test the effectiveness of this transfer program income, applying the Markov chain methods and differences in differences. | Quasi-experimental | Unspecified | 27 federal units | National | Average per capita household income per deciles of the population of the federation units in 1999, 2003, 2005 and 2009. | After the implementation of BFP, the “cycle of poverty” presented signs of rupture, due to a tendency of convergence to higher levels of income and because of the likelihood of mitigating the poorest income classes of the Brazilian population. For people in extreme poverty, there is a likelihood of social improvement equal to 66.7%. Differences in differences results=BFP participating families had an average income above R $ 254.49 in contrast to non-benefited families. |
| Pescarini et al. (2020) | Health | We evaluated the association between the Brazilian Bolsa Familia (BFP) conditional cash transfer program and new leprosy case detection using linked records from 12,949,730 families in the 100 Million Brazilian Cohort (2007–2014) | Propensity score matching; large-scale linked cohort | Information not clear | 12,949,730 families | National | New leprosy case detection. | BFP participation was not associated with leprosy incidence overall (incidence rate ratio (IRR)Poisson = 0.97, 95% CI: 0.90, 1.04) but was associated with lower leprosy incidence when restricted to families living in high-burden municipalities (irrpoisson = 0.86, 95% CI: 0.77, 0.96). In high-burden municipalities, the association was particularly pronounced for paucibacillary cases (irrpoisson = 0.82, 95% CI: 0.68, 0.98) and cases with leprosy-associated disabilities (irrpoisson = 0.79, 95% CI: 0.65, 0.97). |
| Pescarini et al. (2020b) | Health | We estimated the association of the Brazilian conditional cash transfer programme, the Programa Bolsa Família (PBF), on leprosy treatment adherence and cure in patients in Brazil. | Propensity score matching; large-scale linked cohort | All ages | 11,456 new leprosy cases | National | Leprosy treatment adherence and cure. We defined leprosy treatment adherence as achieving the prescribed number of multidrug therapy doses (ie, six or more doses for paucibacillary disease and 12 or more doses for multibacillary disease) and leprosy cure as recorded by health professionals. | Receiving PBF before diagnosis was associated with adherence to treatment and cure. For patients with multibacillary disease, PBF beneficiaries had better treatment adherence and cure than non-beneficiaries. In the propensity score-matched analysis in 2654 children younger than 15 years with leprosy, PBF exposure was not associated with leprosy treatment adherence or cure. |
| Pires (2013) | Education | To ponder on poverty fighting strategies, from the minimum school attendance standpoint, present on the design of the "Programa Bolsa Família (PBF)", a cash transfer program of the Federal Government. | Qualitativa e descritiva | Information not clear | 22 people | Campinas, SP | Symbolic meanings, to some extent unforeseen, of conditionalities and education when taking into account the views of the participants of the BFP. | Respondents do not ignore the possible practical effects of putting and keeping their children in school. However, the interviews suggest that the fulfillment of this condition, in order to receive the benefit of the PBF, also establishes a relationship of exchange and reciprocity between the beneficiaries and the State that may be interesting if we consider the social bonds that derive from this relationship. |
| Pires (2012) | Poverty | To understand the beneficiaries’ perception that the “Family Allowance helps”. The aim was to grasp the meaning of this “help” at two moments, trying to understand: how living in a city like Campinas interferes in the way through which people evaluated the program; and, how the word “help” denotes a gendered incorporation of the money. | Qualitative -Ethnographic investigation | 23–53 | 14 adults | Campinas, SP | The meaning of BFP help. | The meaning of BF help is understood as something that supplements something else, something that serves to aid or boost another income — often larger and from other sources. While exercising these competencies provides unquestionable gains — whether as a consumer or in reinforcing domestic authority and autonomyuse of PBF money. |
| Pires & Jardim (2014) | Education | To analyze the effects of the Bolsa Família Program in the town of Catingueira, Paraíba, Brazil, in relation to the changes related to the expansion of consumption, the schooling of children and child labor within families that have started receiving the benefit. | Qualitative | Adults (mothers) | 22 mothers | Catingueira, paraíba | Changes related to the expansion of consumption, the schooling of children and child labor. | There were expansion of consumption possibilities and prioritization of consumption of goods for children; possibility of staying in school to the detriment of working in the fields, with guaranteed minimum family income. Money transfer ensured that the child did not need to work to complete the household income and could continue his studies. |
| Quadros & Santos (2017) | Teenage pregnancy | To discuss the barriers to obtain reversible contraception and restrictions in the supply of sterilization among BFP beneficiaries in Recife, Pernambuco Brazil | Qualitative- Ethnographic research | 20–19 years | 5 | Coque community, Recife, PE | Access to contraceptive methods and sterilization. | The five women reported obstacles to obtain reversible contraceptive methods via SUS. Their argument that they are beneficiaries of Bolsa Família aims to emphasize their poverty and increase the odds of obtaining sterilization. Only two of the women had succeeded in obtaining sterilization. All claimed poor quality of health and education services. BFP benefit had an impact on households, and it is mainly used to buy food and child`s clothes. |
| Rasella et al. (2013) | Health | To assess the effect of the BFP on deaths of children younger than 5 years (under-5), overall and resulting from specific causes associated with poverty: malnutrition, diarrhoea, and lower respiratory infections. | Mixed ecological design.(multivariable regression analyses of panel data with fixed-effects) | Children <5 years | 2,853 municipalities | National | All-cause under-5 mortality rates=number of deaths of children younger than 5 years per 1000 livebirths. | Under-5 mortality rate, overall and resulting from poverty-related causes, decreased as BFP coverage increased. The increase in BFP coverage increased vaccination coverage for measles, polio, and DPT, reduced the number of pregnant women who delivered without receiving any prenatal care, and reduced rates of under-5 admissions to hospital in a manner much the same as for the reduction in mortality rates, having the strongest eff ect on malnutrition and no eff ect on external causes. |
| Reis-Santos et al. (2019) | Health | To assess the effect of being a beneficiary of a governmental cash transfer program on tuberculosis (TB) treatment cure rates | Quasi-experimental | Adults>18 years | 25,084 adults | Brazil (excluding SP) | TB cure rates. | Being beneficiary of cash transfer improved TB cure rates by 8% in subjects with pulmonary TB. |
| Reynolds (2015) | Education | To evaluate the impact of BFP on attendance of older students provided by the 2008 extension of Bolsa Familia eligibility to 16- and 17-year-olds. | Quasi-experimental | 15–17 | 29,744 adolescents | National | School attendance. | Significant increase in attendance among the 16-year-olds of 6 percentage points, 15 percentage point increase in school attendance for continuously treated, poor, urban boys . No change in attendance for the 17-year-olds who had a gap year in treatment. Secondary result:Youth time use does not change with the conditional cash transfer. |
| Rezende (2018) | Poverty | To discuss effects of the resources of the Bolsa Família Program (BFP) and the Continuous Cash Benefit (CCB) allocated in local social and economic dynamics. | Qualitative | Adults | 4 Charity representatives,  1 religious representative,  10 BFP beneficiaries, 20 BCP beneficiaries | Passa Tempo - MG | The actions developed by private charitable organizations and the buying habits of the families who receive these benefits. | Allocated federal funds on cts have provoked adjustments in the performance of charities to contribute towards reducing the volume of their demands. BFP foment dynamics associated to the consumption of goods and services of a private nature. The existence of non-contributory benefits represents the strengthening of state action in the perspective of social welfare. |
| Rocha et al. (2018) | Poverty | To identify the impact of the PBF on the expenditure value of each category and on the share of expenditure. | Quasi-experimental | Adults (not specified) | 8414 adults | National | Impact of BF on household expendures in food, housing, clothes, transport, health, education, tobacco, personal services, leisure. | The largest impact of the PBF was on food and housing. However, the effect on food was surprisingly negative. On the other hand, the impact on housing was positive. The impact of the PBF is also larger in households where women were the head of household. |
| Rougier et al. (2018) | Poverty | To investigate the impact of BFP on local economic growth | Quasi-experimental | All ages | 184 municipalities of Ceará | Ceará state, Brazil | GDP growth. | It was found a positive and significant local growth impact of the BFP, except for the industrial municipalities where BFP/GDP loses its significant impact on local growth. By contrast, the BFP growth impact is stronger for the non-metropolitan, hinterland and weakly industrialized municipalities than for the whole sample of municipalities. |
| Santos et al. (2020) | Gender equality | We propose a reflection on the transformations that have been operated in the Brazilian social protection, in which they weigh advances and challenges of the dialogue of the Bolsa Família Program with health. | Analysis of 'thematic content' | Information not provided | 29 people | Municipality in the southwestern region of Bahia | Advances and challenges of the interlocution of the Bolsa Família program with health. | The statements materialize advances of the Bolsa Família Program in coping with poverty, especially in situations of material penury, as well as effects on the social recognition and dignity of women beneficiaries. Stigmas and misunderstandings related to the Bolsa Família Program permeate the relationships between beneficiaries and primary health care workers. |
| Santos et al. (2014) | Poverty | To analyze the effectiveness of the Bolsa Família Program (PBF), from the perspective of the beneficiaries of this cash transfer program. | Qualitative-quantitative study | Adults | 50 beneficiaries | Manaus (AM) | Use of benefit in the quality of life, education, health. | The comparison between the period before and after the insertion in the PBF made the beneficiaries report changes related mainly to the increase in income and family consumption capacity. The majority reported that part of the financial resources is used to purchase school supplies and other items, accessories such as clothing and shoes. |
| Santos et al. (2017) | Poverty | To describe the evaluation of mothers who receive a Family Grand (Bolsa Família) living in a riverside community in the Amazon on participation in the program. | Qualitative study | Mothers 26–50 years | 4 mothers | Combú island, municipality of Belém (PA) | A sociodemographic questionnaire and a semi-structured interview were applied to understand how the mothers perceived their life before and after the program. | The mothers no longer perceived themselves as highly dependent on their husband’s income, while the expenditures were realized in a more planned way and the occurrence of situations in which there was no food ceased to exist. The program has a concrete importance in the economic organization of families, providing greater sense of security and stability. |
| Shei et al. (2014) | Health | Examines the impact of Bolsa Família on child health in a slum community in a large urban center. | Cross-sectional | Children < 7 | 567 families (with 1.266 children). 841 (66%) beneficiary and 425 (34%) non-beneficiary children | Salvador, BA | Health status (10 individual items; physical health summary measure; psychosocial health summary measure). | Bolsa Família has increased the odds of children’s visits to the health post for preventive services. Bolsa Família increased their odds for growth monitoring (OR = 2.5; 95% CI 1.3-4.9) and checkups (OR = 1.7; 95% CI 0.9-3.2) and improved psychosocial health (β = 2.6; p = 0.007). |
| Shei (2013) | Health | Examines the impact of the Bolsa Família program on infant mortality rates while accounting for Family Health Program coverage and the potential interaction between the two programs. | A pooled, time-series, cross-sectional design | Preschoolers | Around 23 millions of beneficiary families (coverage of BFP) | National | Municipal-level all-cause infant mortality rate. | Bolsa Família program coverage was associated with a decline in the infant mortality rate. The Bolsa Família program encourages families to seek preventive health care, and the presence of the Family Health Program in a municipality may help beneficiaries meet the Bolsa Família program’s conditionalities. |
| Silva & Paes (2019) | Health | To assess the effect of the PBF on child mortality (CM) in the semiarid region of Brazil during the period of 2004-2010. | Longitudinal ecological study | Children (not clear if <1 year) | 1,133 municipalities | Brazilian semiarid region | Child mortality rate (number of deaths/live births) (does not specify if <1 year). | The PBF and the FHS played significant roles in reducing CM. |
| Silva, Paes & Silva (2018) | Health | To evaluate the impact of the social programs: Bolsa Família Program and Family Health Strategy and fertility on child mortality in the brazilian Semiarid, during the period 2005-2010. | Longitudinal ecological study | Preschoolers | 1,133 municipalities but 8 excluded, totalizing 1,125 municipalities | MA, PI, CE, RN, PB, PE, AL, SE, BA and MG | Infant mortality rate. | The linear regression with fixed effects showed a negative and statistically significant association (p <0.001) between infant mortality and co-variables: Coverage of the Bolsa Família Program, Percentage of beneficiary families of the PBF accompanied by health conditionalities and Coverage of the Family Health Strategy. |
| Silva et al. (2020) | Health | To estimate the effect of being a beneficiary of the Bolsa Família Program (BFP) in the vaccination of children aged 13 to 35 months. | Propensity score matching | Birth (2010) and first follow-up (2011 to 2013), from 13 to 35 months of age. | 532 children | Ribeirão Preto and São Luís | Collected at the time of follow-up, was a “childhood vaccination schedule,” categorized as complete and incomplete. For its construction, the 7 vaccines that must be taken in the first year of life were used as parameters, according to the National Calendar of Vaccination of children of the Brazilian Ministry of Health in force since the beginning of 2010. | Considering a monthly per capita family income of up to R$ 280.00, being a beneficiary of the BFP had no effect on the childhood vaccination schedule, according to weighing by the inverse of the probability of exposure (SL-coefficient: −0.01; 95%CI −0.07 to 0.04; p = 0.725 and RP-coefficient: 0.04; 95%CI −0.02 to 0.10; p = 0.244). |
| Simões & Sabates (2014) | Education | To investigate whether the Brazilian conditional cash transfer programme (BF), contributes to improvements in school performance in test scores and pass-grades, and reductions in dropout rates for 4th grade children | Quasi-experimental design | 4th grade classes | 35,172 schools | National | Schools’ mean tests scores as measured in the national exam – Prova Brasil 2007 – as well as effects on pass-grade and dropout rates. | The proportion of beneficiaries in the school is correlated with more students being promoted and fewer students abandoning school over the academic year. Regarding the performance of schools in Portuguese proficiency by Prova Brasil, a significant and negative impact was found between 2005 to 2007. The performance in mathematics was unsatisfactory in all analyzes, being negative and significant for the year 2007. |
| Simões & Soares (2012) | Teenage pregnancy | To assess whether the Bolsa Família Program contributes to increase the fertility among the beneficiaries, since the increase in family size, up to a certain limit, leads to an increase in the benefits. | Quasi-experimental design | 16–44 years | 7,841 women | National | Fertility rate (number of children born in the period). | The results show that the PBF didn’t increase feritlity rate, at least for the beginning of the program. Rather, the beneficiaries seem to be more inclined to trade quantity for quality than non-beneficiaries eligible for the program. |
| Sperandio et al. (2016) | Poverty | To assess the impact of the Bolsa Família Program on the energy and nutrient intakes of beneficiaries from the Brazilian Northeast and Southeast regions | Quasi-experimental design | >10 years | 5,975 individuals | Northest and Southest Brazilian regions | Using the records of the detailed food intake on two nonconsecutive days of individuals aged more than 10 years were calculated Intakes of energy, protein, total lipids, total carbohydrates, added sugar, fiber, calcium, iron, sodium, zinc, selenium, vitamin B1, vitamin B6, vitamin C, vitamin A, vitamin D, and vitamin E. | The program increased energy and macronutrient intakes and decreased calcium and vitamin A, D, E, and C intakes of adolescent beneficiaries in both regions. Adult beneficiaries from the Southeast region increased their fiber, iron, and selenium intakes, and those from the Northeast region decreased their energy, lipid, added sugar, sodium, zinc, vitamin E, and pyridoxine intakes. |
| Sperandio et al. (2017) | Poverty | To assess and compare the impact of the Bolsa Família Program (Family Allowance) on the nutritional status of children and adolescents from the Brazilian Northeastern and Southeastern regions. | Quasi-experimental design | 5–19 years | 8,328 individuals | Northest and Southest Brazilian regions | Ratios of underweight, stunted, and overweight. | The ratio of underweight children and adolescents was, on average, 1.1% smaller in the beneficiary families than in the non-beneficiary families in the Northeastern region. As for the Southeastern region, the ratio of overweight children and adolescents was, on average, 4.2% smaller in the beneficiary families. The program did not affect stunting in either region. |
| Sperandio et al. (2017b) | Poverty | To evaluate the impact of the Bolsa Família Program (PBF) on food consumption in the northeast and southeast regions of Brazil. | Quasi-experimental design | >10 years | 5,975 individuals | Northest and Southest Brazilian regions | Food Consumption was assessed through two food records. The food was categorized into four groups: fresh or minimally processed food; culinary ingredients; processed food; and ultra-processed food. | In both regions, more than 60% of the daily total calories consumed by PBF recipients came from foods that had not undergone industrial processing. The recipients of PBF had a low level of consumption of processed and ultra-processed food in both regions, and an increased level of consumption of fresh or minimally processed food in the northeast. |
| Tavares (2010) | Employability | To evaluate the impact of the Bolsa Família Program on the decisions of mothers living in beneficiary families with respect to their participation in the market and their working hours. | Propensity score matching | Mean of ages: Rated group (37), Control group 1 (37), Control group 2 (32), Control group 3 (38) | Evaluated group: 15,245; control group 1: 2,532; control group 2: 8,456; control group 3: 31,414 | Campinas, SP | Decisions related to the job offer and Monthly working hours. | Participation in the family scholarship increases participation in the labor market. |
| Testa et al. (2013) | Poverty | To reflect on the contribution of Bolsa Família Program to the confrontation of poverty and an increased autonomy of beneficiary subjects. | Mixed quantitative and qualitative study (Please select the qualitative data for data summary) | Adults | N=103 (survey) N=3 (CRAS professionals) N=4 (interviews with families) | Porto alegre, RS | Autonomy: to identify whether and how the beneficiaries of the BFP have managed to overcome deprivation of liberty about: food, education, health, family relationships, risk (including violence), social life, housing, leisure and work. | Education is the deprivation aspect which obtained the best results in the perception of families in search for autonomy. Participation in social support activities has a direct effect on the perception of family improved situation; however, the participation of families showed to be incipient. |
| Uchimura et al. (2012) | Poverty | To understand the perceptions of participants of the ‘Bolsa Família’ Program on the quality of their food | Qualitative study | >18 years | 38 beneficiaries | Curitiba municipality | Perceptions about their food habits. | Participants reported low variability in dietary intake. They perceive the benefit as a necessary help. The recognition of social vulnerability and consequent feeding habit insecurity to which such groups are subject was the main finding, as well as feelings of resignation. |
| Vasconcelos et al. (2017) | Employability | To analyze the relation between the Bolsa Família Program and the probability that young adults aged from 18 to 29 years belong to “NEET” generation. | Quasi-expetimental design | Brazilians aged 18-29 with per capita household income <280 BRL | 16,196,151 adults | National | Census data on working and educational status. | Bolsa Família Program decreases the probability that young people belong to the “NEET” generation. |

**Supplementary chart 1.** Databases and search strategies used.

| **Database and delimitations** | **Strategy used** |
| --- | --- |
| Education Resources Information Center (ERIC)  Since 2003, peer reviewed only | (“bolsa familia” OR “bolsa família” OR "cash transfer" OR (conditional OR unconditional) ("cash transfer") OR "financial support" OR "poverty reduction scheme" OR "bolsa familia" OR "cash allowance" OR "cash allowances" OR "payment allowance" OR "payment allowances" OR "monetary allowance" OR "monetary allowances" OR "money allowance" OR "money allowances" OR "family allowance" OR "family allowances" OR "cash benefit" OR "cash benefits" OR "payment benefit" OR "payment benefits" OR "monetary benefit" OR "monetary benefits" OR "money benefit" OR "money benefits" OR "alleviation of poverty" OR "anti-poverty policies" OR "anti-poverty policy" OR "financial assistance" OR "financial assistances") AND (brazil) |
| Embase  2003-2021, title or abstract, Embase only (without Medline) | (('cash transfer':ab,ti OR 'monetary assistance':ab,ti OR 'monetary incentive':ab,ti OR 'monetary allowance':ab,ti OR 'monetary benefit':ab,ti OR 'monetary support':ab,ti OR 'financial support':ab,ti OR 'poverty reduction scheme':ab,ti OR 'alleviation of poverty':ab,ti OR 'anti-poverty polic*':ab,ti) AND brazil:ab,ti OR 'bolsa família':ab,ti OR 'bolsa familia':ab,ti) AND [2003-2021]/py |
| National Health Economics Information Portal (ECOS)  2003-2021, title/resume/subject, articles only | (("cash transfer") OR ("monetary assistance") OR ("monetary incentive") OR ("monetary allowance") OR ("monetary benefit") OR ("monetary support") OR ("financial support") OR ("poverty reduction") AND (brazil)) OR ("bolsa familia") |
| PubMed  2003-2021, title/abstract | (("cash transfer"[Title/Abstract] OR "monetary assistance"[Title/Abstract] OR "monetary incentive"[Title/Abstract] OR "monetary allowance"[Title/Abstract] OR "monetary benefit"[Title/Abstract] OR "monetary support"[Title/Abstract] OR "financial support"[Title/Abstract] OR "anti-poverty polic*"[Title/Abstract]) AND "brazil"[Title/Abstract]) OR "bolsa familia"[Title/Abstract] OR "bolsa familia"[Title/Abstract] |
| Science Direct  2003-2021, research articles only; title/abstract or author-specified keywords | ("social assistance" OR "social policy" OR "social welfare" OR "poverty alleviation" OR "poverty reduction" OR "family policy" OR "cash transfer") AND (brazil) OR ("bolsa família") |
| Scientific Electronic Library Online (SciELO)  2003-2021, abstract, articles only | (ab:((((cash transfer) OR (transferência de renda) OR (monetary assistance) OR (assistência monetária) OR (monetary incentive) OR (monetary allowance) OR (monetary benefit) OR (monetary support) OR (financial support) OR (apoio financeiro) OR (poverty reduction scheme) OR (alleviation of poverty) OR (combate à pobreza) OR (anti-poverty polic*)) AND (Brazil OR Brasil)) OR (bolsa família) OR (bolsa familia))) |

**Supplementary chart 2.** Numbering of articles for consultation of the Table 1.

|  | **Reference** |
| --- | --- |
| 1 | ALEJO, Javier; BÉRGOLO, Marcelo; CARBAJAL, Fedora. Las transferencias públicas y su efecto distributivo. La experiencia de los países del Cono Sur en el decenio de los 2000. El trimestre económico, v. 81, n. 321, p. 163-198, 2014. |
| 2 | ALMEIDA, Aléssio Tony Cavalcanti de; MESQUITA, Shirley Pereira de; SILVA, Magno Vamberto Batista da. Impactos do Programa Bolsa Família sobre a diversificação do consumo de alimentos no Brasil. 2016. |
| 3 | Alves H, Escorel S. Processos de exclusão social e iniquidades em saúde: um estudo de caso a partir do Programa Bolsa Família, Brasil. Rev Panam Salud Publica. 2013;34(6):429–36. |
| 4 | ALVES, Flávia Jôse Oliveira; MACHADO, Daiane Borges; BARRETO, Maurício L. Effect of the Brazilian cash transfer programme on suicide rates: a longitudinal analysis of the Brazilian municipalities. Social psychiatry and psychiatric epidemiology, v. 54, n. 5, p. 599-606, 2019. |
| 5 | AMARAL, Ernesto Friedrich de Lima; MONTEIRO, Vinícius do Prado. Avaliação de Impacto das Condicionalidades de Educação do Programa Bolsa Família (2005 e 2009). Dados, v. 56, n. 3, p. 531-570, 2013. |
| 6 | Andrade V. M., Chein F, Rodrigues de Souza L, Puig i Junoy J. Income transfer policies and the impacts on the immunization of children: the Bolsa Família Program. Cad. Saúde Pública. 2012 Jul;28(7):1347-58. |
| 7 | Araujo, Jevuks Matheus de, Alves, Janielle do Amaral, & Besarria, Cássio da Nóbrega. (2013). O impacto dos gastos sociais sobre os indicadores de desigualdade e pobreza nos estados brasileiros no período de 2004 a 2009. Revista de Economia Contemporânea, 17(2), 249-275. https://dx.doi.org/10.1590/S1415-98482013000200003 |
| 8 | BARRIENTOS, Armando; DEBOWICZ, Darío; WOOLARD, Ingrid. Heterogeneity in Bolsa Família outcomes. The Quarterly Review of Economics and Finance, v. 62, p. 33-40, 2016. |
| 9 | Bohn, S., et al. (2014). "Can conditional cash transfer programs generate equality of opportunity in highly unequal societies? Evidence from Brazil." Revista de Sociologia e Política 22(51): 111-133. |
| 10 | Camargo, P. C. and E. T. Pazello (2014). "Uma análise do efeito do programa bolsa família sobre o desempenho médio das escolas brasileiras." Economia Aplicada 18(4): 623-640. |
| 11 | Campoli, J. S., et al. (2020). "The efficiency of Bolsa Familia Program to advance toward the Millennium Development Goals (MDGs): A human development indicator to Brazil." Socio-Economic Planning Sciences 71: 100748. |
| 12 | CARTER, Daniel J. et al. The impact of a cash transfer programme on tuberculosis treatment success rate: a quasi-experimental study in Brazil. BMJ global health, v. 4, n. 1, p. e001029, 2019. |
| 13 | Carvalho, A. T. d., et al. (2014). "Condicionalidades em saúde do programa Bolsa Família – Brasil: uma análise a partir de profissionais da saúde." Saúde e Sociedade 23(4): 1370-1382. |
| 14 | Carvalho, G. R., et al. (2015). "Demand analysis on food: effects of Bolsa Família on dairy consumption as a source of calcium." Planej. polít. públicas(45): 221-244. |
| 15 | Cavalcanti, D. M., et al. (2013). "Programa bolsa família e o nordeste: impactos na renda e na educação, nos anos de 2004 e 2006." Revista de Economia Contemporânea 17(1): 99-128. |
| 16 | Cecchin, H. F. G. and T. G. Parente (2019). "Relações de gênero no Acampamento Ilha Verde: discutindo o (des)empoderamento das mulheres beneficiárias do Bolsa Família." Interações (Campo Grande) 20(3): 907-921. |
| 17 | Cechin, L. A. W., et al. (2015). "O Impacto das Regras do Programa Bolsa Família Sobre a Fecundidade das Beneficiárias." Revista Brasileira de Economia 69(3): 303-329. |
| 18 | Chioda, L., et al. (2016). "Spillovers from conditional cash transfer programs: Bolsa Família and crime in urban Brazil." Economics of Education Review 54: 306-320. |
| 19 | Chitolina, L., et al. (2016). "The Impact of the Expansion of the Bolsa Família Program on the Time Allocation of Youths and Their Parents." Revista Brasileira de Economia 70(2): 183-202. |
| 20 | Coelho, P. L. and A. S. S. d. A. Melo (2017). "Impacto do Programa “Bolsa Família” sobre a qualidade da dieta das famílias de Pernambuco no Brasil." Ciência &amp; Saúde Coletiva 22(2): 393-402. |
| 21 | Correa Junior, C. B., et al. (2019). "Impactos do Programa Bolsa Família no mercado de trabalho dos municípios brasileiros." Revista de Administração Pública 53(5): 838-858. |
| 22 | Costa, R. A., et al. (2018). "Impactos do Programa Bolsa Família no mercado de trabalho e na renda dos trabalhadores rurais." Nova Economia 28(2): 385-416. |
| 23 | Costa-Fernandez, E. and C. A. B. Munoz (2019). "Subjetividades de beneficiárias do Programa Bolsa Família em contexto rural." Fractal: Revista de Psicologia 31(1): 35-42. |
| 24 | DA MOTA, D. M. et al. Extractive family work under the influence of public policies. Revista de Economia e Sociologia Rural, v. 52, n. Suppl. 1, 2014. |
| 25 | De Andrade, K. V. F., et al. (2018). "Effect of Brazil's Conditional Cash Transfer Programme on the new case detection rate of leprosy in children under 15 years old." Leprosy Review 89(1): 13-24. |
| 26 | de Brauw, A., et al. (2014). "The Impact of Bolsa Família on Women’s Decision-Making Power." World Development 59: 487-504. |
| 27 | de Brauw, A., et al. (2015). "The Impact of Bolsa Família on Schooling." World Development 70: 303-316. |
| 28 | de Senna, V. and A. M. Souza (2016). "Assessment of the relationship of government spending on social assistance programs with Brazilian macroeconomic variables." Physica A: Statistical Mechanics and its Applications 462: 21-30. |
| 29 | De Souza, R. A., et al. (2018). "Family health and conditional cash transfer in Brazil and its effect on tuberculosis mortality." International Journal of Tuberculosis and Lung Disease 22(11): 1300-1306. |
| 30 | DUARTE, Gisléia Benini; SAMPAIO, Breno; SAMPAIO, Yony. Bolsa Família Program: impact of transfers on food expenditure in rural families. Rev. Econ. Sociol. Rural, Brasília , v. 47, n. 4, p. 903-918, dez. 2009. |
| 31 | Dyngeland, C., et al. (2020). "Assessing multidimensional sustainability: Lessons from Brazil’s social protection programs." Proceedings of the National Academy of Sciences of the United States of America 117(34): 20511-20519. |
| 32 | Faria, M. V. B. L., et al. (2020). "Programa Bolsa Família como estrategia de diversificação dos meios de vida rurais: uma experiência no sul de Minas Gerais." Revista de Economia e Sociologia Rural 58(3). |
| 33 | Garmany, J. (2016). "Neoliberalism, governance, and the geographies of conditional cash transfers." Political Geography 50: 61-70. |
| 34 | Glewwe, P, Kassouf, A. (2012).The impact of the Bolsa Escola/Familia conditional cash transfer program on enrollment, dropout rates and grade promotion in Brazil. Journal of Development Economics, 97(2): 505-517. |
| 35 | Guanais F. C. (2015). The combined effects of the expansion of primary health care and conditional cash transfers on infant mortality in Brazil, 1998-2010. *American journal of public health*, *105 Suppl 4*(Suppl 4), S593–S592. https://doi.org/10.2105/AJPH.2013.301452r |
| 36 | Hecktheuer, P. A., Souza, C. B. C., & Hecktheuer, F. R. (2018). Desenvolvimento como Liberdade em uma Comunidade Ribeirinha da Amazônia: uma análise dos efeitos do Programa Bolsa Família em São Carlos, Porto Velho, Rondônia. *Sequência (Florianópolis)*, *78*, 119–148. https://doi.org/10.5007/2177-7055.2018v39n78p119 |
| 37 | Hone, T., et al. (2019). "Effect of economic recession and impact of health and social protection expenditures on adult mortality: a longitudinal analysis of 5565 Brazilian municipalities." The Lancet Global Health 7(11): e1575-e1583. |
| 38 | Hone, T., et al. (2020). "Primary healthcare expansion and mortality in Brazil’s urban poor: A cohort analysis of 1.2 million adults." PLoS Medicine 17(10). |
| 39 | LABRECQUE, Jeremy A. et al. Effect of a conditional cash transfer program on length-for-age and weight-for-age in Brazilian infants at 24 months using doubly-robust, targeted estimation. Social Science & Medicine, v. 211, p. 9-15, 2018. |
| 40 | Lima, F., et al. (2017). A territorialização do programa Bolsa Família na comunidade rural Sítio Carnaubal - Água Nova/RN: a voz dos beneficiários. GOT, Revista de Geografia e Ordenamento do Território(12): 179-203. |
| 41 | Litwin, A., et al. (2019). A conditional cash transfer and Women's empowerment: Does Bolsa Familia Influence intimate partner violence? Social Science and Medicine 238. |
| 42 | Machado, D. B., et al. (2018). "Conditional cash transfer programme: Impact on homicide rates and hospitalisations from violence in Brazil." PLoS One 13(12): e0208925. |
| 43 | Magalhães, K. A., et al. (2011). "Entre o conformismo e o sonho: percepções de mulheres em situação de vulnerabilidade social à luz das concepções de Amartya Sen." Physis: Revista de Saúde Coletiva 21(4): 1493-1514. |
| 44 | MARIANO, Silvana Aparecida; CARLOTO, Cássia Maria. Aspectos diferenciais da inserção de mulheres negras no Programa Bolsa Família. Soc. estado., Brasília , v. 28, n. 2, p. 393-417, ago. 2013. |
| 45 | Marins, Mani Tebet Azevedo de. (2018). O ‘feminino’ como gênero do desenvolvimento. Revista Estudos Feministas, 26(1), e39010. Epub January 15, 2018. |
| 46 | Marins, M. T. (2014). REPERTÓRIOS MORAIS E ESTRATÉGIAS INDIVIDUAIS DE BENEFICIÁRIOS E CADASTRADORES DO BOLSA FAMÍLIA. *Sociologia &amp; Antropologia*, *4*(2), 543–562. https://doi.org/10.1590/2238-38752014v4210 |
| 47 | Martins, A.P.B., Monteiro, C.A. Impact of the Bolsa Família program on food availability of low-income Brazilian families: a quasi experimental study. BMC Public Health 16, 827 (2016). |
| 48 | Mattar, V., Monteiro, R. de A., & Azize, R. L. (2021). Vulnerabilidade e precariedade em uma favela carioca: ruídos, controles e convenções a partir do Programa Bolsa Família. *Ciência &amp; Saúde Coletiva*, *26*(9), 4253–4262. https://doi.org/10.1590/1413-81232021269.18042020 |
| 49 | Mello, L. M. M. J. d. (2020). "O Bolsa Família e seus impactos sobre a inserção laboral de beneficiários no município de São Luís." Serviço Social &amp; Sociedade(137): 113-134. |
| 50 | MELO, Raul da Mota Silveira; DUARTE, Gisléia Benini. Impacto do Programa Bolsa Família sobre a frequência escolar: o caso da agricultura familiar no Nordeste do Brasil. Rev. Econ. Sociol. Rural, Brasília, v. 48, n. 3, p. 635-657, 2010. |
| 51 | MOREIRA, Gustavo Carvalho et al. Programa Bolsa Família e violência doméstica contra a mulher no Brasil. Estud. Econ., São Paulo, v. 46, n. 4, p. 973-1002, dez. 2016. |
| 52 | Moreira, Nathalia Carvalho, Ferreira, Marco Aurélio Marques, Lima, Afonso Augusto Teixeira de Freitas Carvalho, & Ckagnazaroff, Ivan Beck. (2012). Empoderamento das mulheres beneficiárias do Programa Bolsa Família na percepção dos agentes dos Centros de Referência de Assistência Social. Revista de Administração Pública, 46(2), 403-423. |
| 53 | Morton, G. (2019). The power of lump sums: Using maternity payment schedules to reduce the gender asset gap in households reached by Brazil’s Bolsa Família conditional cash transfer. World Development, 113, 352-367. |
| 54 | NANES, Giselle; QUADROS, Marion Teodósio de. Bolsa Família Program, Job Market and Women Recipient Agency in Coque (Recife-PE). Cad. Pagu, Campinas, n. 52, e185209, 2018. |
| 55 | Nascimento, Elcio Costa, Carvalho, João Paulo Leão de, Cruz, Benedito Ely Valente da, & Calvi, Miqueias Freitas. (2017). O papel do Programa Bolsa Família na segurança alimentar das famílias do Território do Marajó, PA. Interações (Campo Grande), 18(2), 59-70. |
| 56 | NERI, Marcelo; CAMILLO OSORIO, Manuel. Bolsa Família, time spent in school and students’ motivations. Rev. Adm. Pública, Rio de Janeiro, v. 53, n. 5, p. 859-878, Sept. 2019. |
| 57 | NERY, Joilda Silva et al. Effect of Brazil's conditional cash transfer programme on tuberculosis incidence. The international journal of tuberculosis and lung disease, v. 21, n. 7, p. 790-796, 2017. |
| 58 | NERY, Joilda Silva et al. Effect of the Brazilian conditional cash transfer and primary health care programs on the new case detection rate of leprosy. PLoS Negl Trop Dis, v. 8, n. 11, p. e3357, 2014. |
| 59 | Nunes, J. A. and J. L. Mariano (2015). "Efeitos dos Programas de Transferência de Renda sobre a Oferta de Trabalho Não Agrícola na Área Rural da Região Nordeste." Revista de Economia e Sociologia Rural 53(1): 71-90. |
| 60 | OLIOSI, Janaina Gomes Nascimento et al. Effect of the Bolsa Familia Programme on the outcome of tuberculosis treatment: a prospective cohort study. The Lancet global health, v. 7, n. 2, p. e219-e226, 2019. |
| 61 | Olson, Z., Gardner Clark, R., & Reynolds, S. A. (2018). Can a Conditional Cash Transfer Reduce Teen Fertility? The Case of Brazil’s Bolsa Familia. Journal of Health Economics. |
| 62 | Passos, L. and F. Waltenberg (2016). "Bolsa Família e assimetrias de gênero: reforço ou mitigação?" Revista Brasileira de Estudos de População 33(3): 517-539. |
| 63 | Peixoto, J. B. de S., Silva, V. C. da, Paiva, A. de A., & Gama, J. S. da F. A. (2011). Significado das condicionalidades do programa bolsa família para beneficiários acompanhados numa estratégia saúde da família em Campina Grande – PB. *Rev. bras. ciênc. saúde*, *15*(2). http://periodicos.ufpb.br/index.php/rbcs/article/view/8211 |
| 64 | Peixoto, J. B. de S., Silva, V. C. da, Paiva, A. de A., & Gama, J. S. da F. A. (2011). Transformações sobre as condições de vida de beneficiários do programa bolsa família acompanhados em unidade básica de saúde da família de Campina Grande, Paraíba. *Rev. baiana saúde pública*, *35*(3). http://files.bvs.br/upload/S/0100-0233/2011/v35n3/a2637.pdf |
| 65 | Peña, C. R., et al. (2015). "A eficácia das transferências de renda: as tendências da desigualdade antes e depois do Programa Bolsa Família." Revista de Administração Pública 49(4): 889-914. |
| 66 | Pescarini, J. M., et al. (2020). "Conditional Cash Transfer Program and Leprosy Incidence: Analysis of 12.9 Million Families from the 100 Million Brazilian Cohort." American Journal of Epidemiology 189(12): 1547-1558. |
| 67 | Pescarini, J. M., et al. (2020). "Effect of a conditional cash transfer programme on leprosy treatment adherence and cure in patients from the nationwide 100 Million Brazilian Cohort: a quasi-experimental study." The Lancet Infectious Diseases 20(5): 618-627. |
| 68 | Pires, A. (2013). Afinal, para que servem as condicionalidades em educação do Programa Bolsa Família? *Ensaio: Avaliação e Políticas Públicas em Educação*, *21*(80), 513–531. https://doi.org/10.1590/S0104-40362013000300007 |
| 69 | Pires, A. (2012). "Orçamento familiar e gênero: percepções do Programa Bolsa Família." Cadernos de Pesquisa 42(145): 130-161. |
| 70 | Pires, F. F. and G. A. d. S. Jardim (2014). "Geração bolsa família escolarização, trabalho infantil e consumo na casa sertaneja (Catingueira/PB)." Revista Brasileira de Ciências Sociais 29(85): 99-112. |
| 71 | Quadros, M. T. and G. Santos (2017). "Barriers to the search for female sterilization among women in the Bolsa Familia Program." Cad Saude Publica 33(4): e00152515. |
| 72 | Rasella, D., et al. (2013). "Effect of a conditional cash transfer programme on childhood mortality: A nationwide analysis of Brazilian municipalities." The Lancet 382(9886): 57-64. |
| 73 | Reis-Santos, B., et al. (2019). "Tuberculosis in Brazil and cash transfer programs: A longitudinal database study of the effect of cash transfer on cure rates." PLoS One 14(2). |
| 74 | Reynolds, S. A. (2015). "Brazil's Bolsa Familia: Does it work for adolescents and do they work less for it?" Economics of Education Review 46: 23-38. |
| 75 | Rezende, A. d. F. (2018). "OS BENEFÍCIOS NÃO CONTRIBUTIVOS EM DINÂMICAS SOCIAIS E ECONÔMICAS LOCAIS: ESTUDO DE CASO EM PASSA TEMPO (MG)." Revista Brasileira de Ciências Sociais 33(96). |
| 76 | Rocha, M. A., et al. (2018). "Influência do Programa Bolsa Família na alocação de recursos: uma análise considerando a presença de mulheres no domicílio." Economia e Sociedade 27(3): 997-1028. |
| 77 | Rougier, E., et al. (2018). "The “Local Economy” Effect of Social Transfers: An Empirical Assessment of the Impact of the Bolsa Família Program on Local Productive Structure and Economic Growth." World Development 103: 199-215. |
| 78 | Santos, A. M. d., et al. (2020). "Tensões e contradições da proteção social na fronteira da assistência com a saúde." Trabalho, Educação e Saúde 18(2). |
| 79 | Santos, M. C. M. d., et al. (2014). "A voz do beneficiário: uma análise da eficácia do Programa Bolsa Família." Revista de Administração Pública 48(6): 1381-1405. |
| 80 | Santos, T. M. d., et al. (2017). "Avaliação de Beneficiárias Ribeirinhas da Amazônia sobre o Programa Bolsa Família." Psicologia: Teoria e Pesquisa 33. |
| 81 | Shei et al. (2014). The impact of Brazil’s Bolsa Família conditional cash transfer program on children’s health care utilization and health outcomes. BMC International Health and Human Rights. 14:10. |
| 82 | Shei, A. (2013). "Brazil's conditional cash transfer program associated with declines in infant mortality rates." Health Affairs 32(7): 1274-1281. |
| 83 | Silva, E. and N. A. Paes (2019). "Bolsa Familia Programme and the reduction of child mortality in the municipalities of the Brazilian semiarid region." Cien Saude Colet 24(2): 623-630. |
| 84 | Silva, E. S. de A. da, Paes, N. A. and Silva, C. C. da. (2018). Effects of government programs and fertility on child mortality in the brazilian Semiarid region. Saúde em Debate. 42(116), 138-147. |
| 85 | Silva, F. S., et al. (2020). "Bolsa Familia program and incomplete childhood vaccination in two Brazilian cohorts." Rev Saude Publica 54: 98. |
| 86 | Simões, A. A. and R. Sabates (2014). "The contribution of Bolsa Família to the educational achievement of economically disadvantaged children in Brazil." International Journal of Educational Development 39: 141-156. |
| 87 | Simões, P. and R. B. Soares (2012). "Efeitos do Programa Bolsa Família na fecundidade das beneficiárias." Revista Brasileira de Economia 66(4): 445-468. |
| 88 | Sperandio, N., et al. (2016). "Impact of the Bolsa Família Program on energy, macronutrient, and micronutrient intakes: Study of the Northeast and Southeast." Revista de Nutrição 29(6): 833-844. |
| 89 | Sperandio, N., et al. (2017). "Impact of Bolsa Família Program on the nutritional status of children and adolescents from two Brazilian regions." Revista de Nutrição 30(4): 477-487. |
| 90 | Sperandio, N., et al. (2017). "The impact of the Bolsa Familia Program on food consumption: a comparative study of the southeast and northeast regions of Brazil." Cien Saude Colet 22(6): 1771-1780. |
| 91 | Tavares, Priscilla Albuquerque. The impact of the Bolsa Família Program on the labor supply of working mothers. (2010). Economia e Sociedade, 19(3), 613-635. |
| 92 | Testa, M. G., et al. (2013). "Análise da contribuição do Programa Bolsa Família para o enfrentamento da pobreza e a autonomia dos sujeitos beneficiários." Revista de Administração Pública 47(6): 1519-1541. |
| 93 | Uchimura, K. Y., et al. (2012). "Quality of food: Perceptions of 'Bolsa Familia' program participants." Ciencia e Saude Coletiva 17(3): 687-694. |
| 94 | Vasconcelos, A. M., et al. (2017). "Programa Bolsa Família e Geração Nem-Nem: Evidências para o Brasil." Revista Brasileira de Economia 71(2): 233-257. |
